# Co-occurrence of memory impairment and fatigue distinguishes post COVID from pandemic-related health effects in the 4-year CON-VINCE cohort study

**DOI:** 10.1038/s41598-025-19984-7

**Published:** 2025-10-27

**Authors:** Patricia Martins Conde, Dmitry Bulaev, Armin Rauschenberger, Jochen Ohnmacht, Joëlle V. Fritz, Marc P. O’Sullivan, François Ancien, Soumyabrata Ghosh, Olena Tsurkalenko, Alexey Kolodkin, Venkata Satagopam, Michel Vaillant, Jochen Klucken, Rejko Krüger, CON-VINCE/ORCHESTRA Study Consortium, Tamir Abdelrahman, Geeta Acharya, Gloria Aguayo, Pinar Alper, Wim Ammerlaan, François Anciens, Ariane Assele-Kama, Christelle Bahlawane, Katy Beaumont, Nadia Beaupain, Lucrèce Beckers, Camille Bellora, Fay Betsou, Luc Biver, Sandie Boly, Dirk Brenner, Henry-Michel Cauchie, Eleftheria Charalambous, Emilie Charpentier, Estelle Coibion, Sylvie Coito, Delphine Collart, Manuel Counson, Brian De Witt, Antonelle Di Pasquale, Olivia Domingues, Claire Dording, Jean-Luc Dourson, Bianca Dragomir, Tessy Fautsch, Jean-Yves Ferrand, Thibault Ferrandon, Ana Festas Lopes, Guillaume Fournier, Joëlle Véronique Fritz, Manon Gantenbein, Piotr Gawron, Laura Georges, Soumyabrata Ghosh, Stéphane Gidenne, Enrico Glaab, Clarissa Gomes, Borja Gomez Ramos, Vyron Gorgogietas, Jérôme Graas, Valentin Groues, Wei Gu, Gaël Hamot, Anne-Marie Hanff, Maxime Hansen, Linda Hansen, Lisa Hefele, Laurent Heirendt, Ahmed Hemedan, Estelle Henry, Margaux Henry, Eve Herkenne, Sascha Herzinger, Christiane Hilger, Laetitia Huiart, Alexander Hundt, Judith Hübschen, Gilles Iserentant, Philipp Jägi, Anne Kaysen, Piyapong Khurmin, Fédéric Klein, Tommy Klein, Stéphanie Kler, Alexey Kolodkin, Rejko Krüger, Pauline Lambert, Jacek Jaroslaw Lebioda, Sabine Lehmann, Marie Leick, Anja Leist, Morgane Lemaire, Andrew Lumley, Annika Lutz, João Manuel Loureiro, Monica Marchese, Tainà Marques, François Massart, Patrick May, Maura Minelli, Alessandra Mousel, Maeva Munsch, Sophie Mériaux, Friedrich Mühlschlegel, Mareike Neumann, Trang Nguyen, Beatrice Nicolai, Marc Paul O’Sullivan, Leslie Ogorzaly, Jochen Ohnmacht, Christiane Olesky, Markus Ollert, Claire Pauly, Laure Pauly, Lukas Pavelka, Christian Penny, Magali Perquin, Achilleas Pexaras, Palma di Pinto, Marie France Pirard, Jean-Marc Plesseria, Guilherme Ramos Meyers, Armin Rauschenberger, Lucie Remark, Antonio Rodriguez, Basile Rommes, Kirsten Rump, Estelle Sandt, Bruno Santos, Venkata P. Satagopam, Aurélie Sausy, Margaux Schmitt, Christiane Schmitt, Reinhard Schneider, Valerie Schröder, Serge Schumacher, Alexandra Schweicher, Sneeha Seal, Jean-Yves Servais, Florian Simon, Amna Skrozic, Chantal Snoeck, Kate Sokolowska, Lara Stute, Hermann Thien, Stéphane Toll, Noua Toukourou, Christophe Trefois, Johanna Trouet, Nguyen Trung, Jonathan Turner, Michel Vaillant, Daniela Valoura Esteves, Carlos Vega Moreno, Charlène Verschueren, Maharshi Vyas, Claus Vögele, Cécile Walczak, Xinhui Wang, Femke Wauters, Bernard Weber, Emilie Weibel, Tania Zamboni, Angelo Ferrari, Dmitry Bulaev, Maud Theresine, Robert Sega, Sabrina Saracino, Amy Parrish, Alexia Mendibide, Anna Schritz, Carlos Gamio, Carolina Coimbra, Clair Meek, Ekaterina Soboleva, François Neu, Gessica Contesotto, Guilherme Marques, Guillaume Nieser, Ilsé Richard, Mesele Valenti, Myriam Alexandre, Myriam Menster, Nassera Aouali, Olena Tsurkalenko, Olga Kofanova, Olivia Roland, Saïda Mtimet, Sonia Lamine, Valéry Bocquet, Victoria Lorentz

**Affiliations:** 1 Luxembourg Centre for Systems Biomedicine (LCSB), University of Luxembourg, Esch-Sur-Alzette, Luxembourg; 2 Luxembourg Institute of Health, Strassen, Luxembourg; 3 Eurostat, European Commission, Luxembourg City, Luxembourg; 4 Centre Hospitalier de Luxembourg, Luxembourg, Luxembourg; 5 Laboratoire National de Santé, Dudelange, Luxembourg; 6 TNS-ILRES, Bertrange, Luxembourg; 7 Luxembourg Institute of Science and Technology, Luxembourg, Luxembourg; 8 Ketterthill, Belvaux, Luxembourg; 9 BioNeXt, Leudelange, Luxembourg; 10 Laboratoire Réunis, Junglinster, Luxembourg; 11 University of Luxembourg, Esch-sur-Alzette, Luxembourg

**Keywords:** Cohort, Post COVID syndrome, Long COVID, PASC, SARS-CoV-2, Long COVID mimics, Viral infection, Epidemiology, Risk factors, Signs and symptoms

## Abstract

A major challenge in diagnosing post COVID lies in differentiating symptoms following a confirmed SARS-CoV-2 infection from those that may also occur in uninfected individuals (post COVID mimics) and be associated with a broader impact of the pandemic. The WHO post COVID definition was applied to the Luxembourgish longitudinal CON-VINCE cohort, where SARS-CoV-2 infection was confirmed via either a positive RT-qPCR or a serology test. Risk factor analysis was conducted on 1,865 individuals. Female gender, lower resilience, greater loneliness, and a higher number of comorbidities were associated with symptoms persistence. The symptomatology and comorbidity profiles of 559 participants (including 50 post COVID and 66 post COVID mimics) were investigated. Two distinct clusters of persistent symptoms were identified: (1) depression with anxiety, present in both infected and non-infected groups, and (2) memory impairment with fatigue, unique to the post COVID group. Therefore, presence of both memory impairment and fatigue may help differentiate post COVID patients from post COVID mimics. Yet, verification that memory impairment was newly developed was not possible, as this symptom was not recorded at baseline. Our findings suggest that future studies should consider factors affecting development of persistent post COVID-like symptoms observed in individuals that were never infected.

**Supplementary Information:**

The online version contains supplementary material available at 10.1038/s41598-025-19984-7.

## Introduction

Coronavirus disease 2019 (COVID-19), caused by the severe acute respiratory syndrome coronavirus 2 (SARS-CoV-2) was the most devastating outbreak of an infectious disease of the twenty-first century causing a major impact on public and mental health, economy and politics. The rapid rise in the number of COVID-19 cases demonstrated the high contagiousness and human-to-human transmission of SARS-CoV-2^1,2^. After several months into the pandemic, healthcare professionals observed that in some patients symptoms persisted even after clearance of the virus^3^.

### Post COVID condition

Persisting symptoms following SARS-CoV-2 infection referred to as "long or post COVID" or "post-acute sequelae of SARS-CoV-2 infection”^4^ have been reported^5^, with approximately 10 to 20% of SARS-CoV-2 infected patients experiencing lingering symptoms following the acute infection phase^6^. However, these numbers vary depending on the study population and the post COVID definition considered.

Since September 2020 the 10th International Classification of Diseases (ICD-10) lists the post COVID-19 condition, whose definition has been updated several times. The World Health Organisation (WHO) has developed a clinical case definition as “post COVID-19 condition occurs in individuals with a history of probable or confirmed SARS-CoV-2 infection, usually 3 months from the onset, if symptoms last for at least 2 months and cannot be explained by an alternative diagnosis”^6,7^. Additionally, other definitions aiming to capture the long-term consequences of SARS-CoV-2 infection have been proposed elsewhere^8^.

Extensive research has been conducted to understand the characteristics, frequency and nature of persisting symptoms after the recovery from an acute infection. The most common reported symptoms at 6 months after infection are fatigue, post-exertional malaise and neurocognitive impairment^8–11^. Furthermore, several risk factors of post COVID have been identified, such as being of female gender or older age, suffering from obesity, depression, anxiety or from a post-traumatic stress disorder^12^. However, the estimated prevalence of post COVID varies widely and depends on the definition and the selection of the timeframe between the infection and the assessment. Furthermore, most of the existing cohort studies include either only confirmed COVID-19 cases e.g. after hospitalisation or rely solely on the participants’ self-reported infection, factors that can introduce bias in the estimated prevalence of post COVID^13,14^.

### Clinical characteristics

The clinical presentation of post COVID might be similar to the chronic fatigue syndrome^13,15^ often triggered by a viral infection as seen in post-Ebola syndrome, chikungunya and in myalgia encephalomyelitis, and has been previously referred as "post-viral fatigue". Persisting symptoms months after viral infection have been also described for other respiratory pathogens, such as Influenza (H1N1) and Middle East respiratory syndrome coronavirus (MERS-CoV)^16^. Individuals suffering from post COVID can present a combination of symptoms that may vary over time, whereas almost any system of a human body can be impacted.

### Challenges in distinguishing post-viral symptoms from pandemic-related health effects

A significant challenge in the diagnosis of post COVID is the differentiation between symptoms specifically caused by SARS-CoV-2 infection from similar symptoms that may develop due to other causes, as many of these symptoms can also occur in individuals who have never been infected^17^. The pandemic itself has created conditions that may lead to the development of persistent symptoms independently of viral infection. Extended lockdowns, social isolation, economic stress, and healthcare disruptions might have impacted both physical and mental health. These pandemic-related stressors could cause or exacerbate symptoms that mirror those reported in post COVID^18,19^. Fatigue, sleep disorders, anxiety, and depression were prevalent before the pandemic and may have increased during it, making it difficult to attribute their presence solely to SARS-CoV-2 infection. Finally, lack of specific biomarkers for post COVID means that post COVID diagnosis often relies on symptom reporting and exclusion of other causes^13,14^. Without objective measures, distinguishing between infection-related and pandemic-related symptoms becomes particularly challenging.

Any research addressing these challenges would require the inclusion of appropriate control groups of uninfected individuals, a careful consideration of pre-existing conditions and pandemic-related stressors. Moreover, more objective diagnostics criteria and a long-term follow-up to better understand disease trajectories would be required. Due to the unique design of the population-based cohort of the Luxembourgish population (CON-VINCE study), which enrolled participants at the onset of the pandemic and since then regularly followed, we had the opportunity not only to identify which persisting symptoms or set of symptoms can be specifically associated with post COVID following a confirmed SARS-CoV-2 infection, but we could also characterise individuals that were never SARS-CoV-2 infected, yet presenting with similar symptoms. Additionally, we further investigated to which extent existing comorbidities could influence the development of new persistent symptoms.

## Methods

### Study design

The CON-VINCE study (COvid-19 National survey for assessing VIral spread by Non-affected CarriErs)^20^ is a longitudinal observational study, where participants (18–84 years) completed online questionnaires, and donated blood and other biosamples, over seven visits between April 2020 and June 2021, n = 1,865 at baseline visit. Serological analyses of blood samples and RT-qPCR (quantitative reverse transcription polymerase chain reaction) from naso/oropharyngeal swabs were performed.

A subset of individuals from the CON-VINCE cohort (n = 1,220) was further followed-up between December 2021 and March 2024 within the framework of ORCHESTRA Europe^21^, where additional online questionnaire data and dried blood samples were collected for four additional visits (CON-VINCE visits 7 to 10).

In total, this manuscript considers the data from 10 study visits, covering the pandemic and lockdown conditions, as well as post-pandemic timepoints. The exact visit dates and visit labels are provided in Supplementary Fig. 1.

### Infection status definition

SARS-CoV-2 infection status was considered positive, if an individual met one or more of the following criteria:

- Positive RT-qPCR result, and/or,
- Positive IgG-S titres to SARS-CoV-2 prior to vaccination, and/or,
- Presence of IgG-N titres to SARS-CoV-2.

The detailed description of the assay kits used in serological and RT-qPCR testing is provided elsewhere^20^.

From the first time point where one or more of these criteria were met, an individual was allocated to the “infected” group until the end of the study.

The “non-infected” group consists of individuals that were never infected, the status of which is confirmed by information from questionnaires and biological analyses of nasal swabs and serum samples, such as negative IgG-N and negative IgG-S before vaccination, at all preceding visits.

### Defining persisting symptoms according to WHO post COVID definition

To assess persistence of symptoms in the context of post COVID, the WHO definition “*continuation or development of new symptoms 3 months after the initial SARS-CoV-2 infection, with these symptoms lasting for at least 2 months with no other explanation”* was implemented^6^.

In this study, this definition was adopted by applying each of the following criteria:

1. Symptoms assessed at 91 or more days since infection [time of assessment]
2. At least 1 symptom “still unresolved” at visit N, and, also at visit N-1 [persistence]
3. Number of days between visits N and N-1 is larger or equal to 60 [persistence]
4. Symptom not present at the baseline visit [newly developed symptom]

Criteria 2–4 were applied irrespective of the infection status, and allowed to identify non-infected individuals who reported persistent and newly developed symptoms defined as “post COVID mimics”.

Most of the symptoms (22 symptoms) were adjusted for their presence at baseline visit (visit 0 of CON-VINCE). For arrhythmia/palpitations, sleep disorders, loss of appetite, and hair loss, the visit used for adjustment for presence at baseline was visit 5 of CON-VINCE, as it was the first visit where these symptoms were assessed. Memory impairment symptom was only assessed from CON-VINCE visit 7 onwards, and hence was not adjusted for its presence at baseline.

Depression and anxiety symptoms were derived from continuous severity scores obtained from the Center for Epidemiologic Studies Depression scale (CES-D)^22,23^ and Generalised Anxiety Disorder 7-item scale (GAD-7)^24,25^. Dichotomisation was conducted as follows:

- CES-D ≥ 16 indicating “Depression-yes”
- GAD-7 ≥ 5 indicating “Anxiety-yes”.

**Fig. 1:**
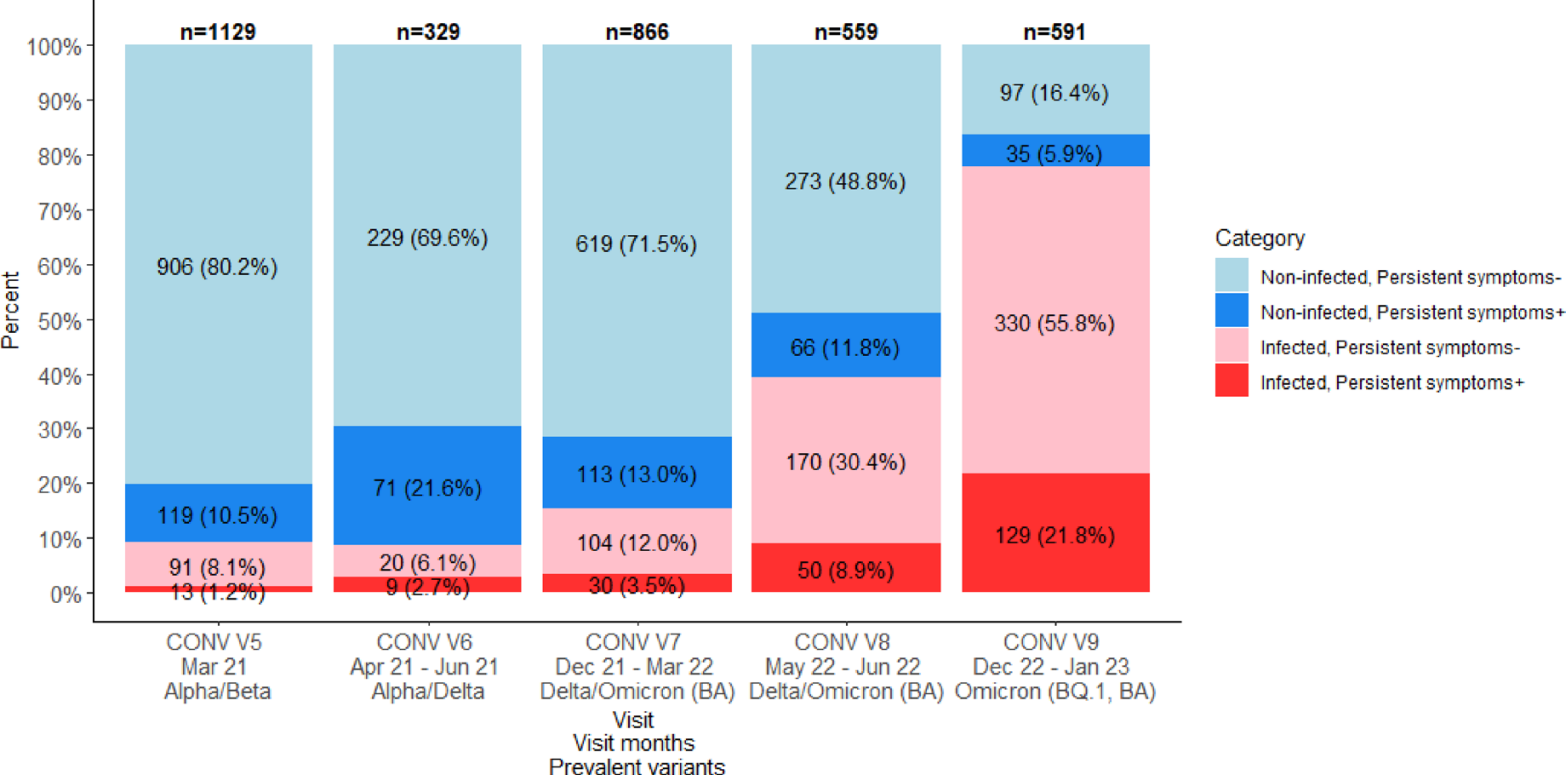
Evolution of the distribution of participants by infection status and by presence of persistent symptoms at different study visits. Most prevalent SARS-CoV-2 variants were obtained from Our World in Data website and based on GISAID datasets^28^.

The complete list of symptoms and comorbidities considered in this manuscript is provided in Supplementary Table 1, and an overview of the distribution of individuals, stratified by SARS-CoV-2 infection status and persistence of symptoms at the different visits of the CON-VINCE study, can be found in Fig. 1 and Supplementary Table 2.

### Statistical analysis

Visit 8 of CON-VINCE was selected for the main analysis, as at this visit the distribution by infection status among all study visits was the most balanced. This allowed for the largest sample size, theoretically ensuring the highest statistical power for analyses (Supplementary Table 2). All analyses were performed in R (version 4.4.1).

### Descriptive statistics

Discrete and continuous variables that had a distribution close to normal (assessed by investigating the histogram) were summarised by the mean and the standard deviation (SD). Non-normal continuous variables were described with a median, lower quartile and upper quartile [Q1, Q3]. Categorical variables were summarised as frequencies with proportions (Table 1).

**Table 1:** Demographic characteristics of the cohort at CON-VINCE visit 8, stratified by SARS-CoV-2 infection status and post COVID status. The number of comorbidities is based on the data recorded at the baseline visit 0.

Risk factor	SARS-CoV-2 infection+		SARS-CoV-2 infection−		Total (row-wise)
Persistent symptoms	+ (post COVID)	−	+ (post COVID mimics)	−	+/-
N	50	170	66	273	559
Gender: Female-N (% column-wise)	29 (58.0%)	75 (44.1%)	39 (59.1%)	139 (50.9%)	282 (50.4%)
Gender: Male-N (% column-wise)	21 (42.0%)	93 (54.7%)	27 (40.9%)	132 (48.4%)	273 (48.8%)
Gender: Diverse/Unknown-N (% column-wise)	0 (0%)	2 (1.2%)	0 (0%)	2 (0.7%)	4 (0.7%)
Age-mean (SD)	50.4 (14.5)	49.6 (14.3)	54.1 (14.0)	57.7 (14.0)	54.2 (14.6)
0 comorbidities-N (% column-wise)	20 (40.0%)	80 (47.0%)	18 (27.3%)	108 (39.6%)	226 (40.4%)
≥ 1 comorbidities - N (% column-wise)	30 (60.0%)	90 (53.0%)	48 (72.7%)	165 (60.4%)	333 (59.6%)
BRS score (resilience)-Median [Q1, Q3]	3.7 [3.0, 4.0]	4 [3.5, 4.3]	3.2 [2.7, 3.7]	4 [3.5, 4.3]	3.8 [3.2, 4.2]
UCLA score (loneliness)-Median [Q1, Q3]	4 [4, 6]	3 [3, 4]	4 [4, 6]	3 [3, 4]	4 [3, 5]

Participants, stratified by infection status, were clustered together based on the presence of symptoms at visit 8 of CON-VINCE and presence of comorbidities at baseline (visit 0) using the *biclust* library in R (Supplementary Table 3). BCBimax was selected as the biclustering method enabling to find submatrices of ones in the binary matrix.

### Inferential statistics

The significance threshold was set to 5% in all analyses. Multiple testing adjustment was conducted to control the false discovery rate (FDR), with the level set at 5%. All tests were two-sided.

The mean age was compared between post COVID and post COVID mimics groups via a Welch’s t-test (normally distributed continuous variable), whereas gender and proportion of individuals with comorbidities were compared via Fisher’s exact tests (categorical variables). The difference in resilience and loneliness between the post COVID and post COVID mimics was assessed via Mann–Whitney tests (non-normally distributed discrete variables) applied to the Brief Resilience Scale (BRS) scores (1–5 range)^26^ and to the University of California Los Angeles (UCLA) 3-item Loneliness Scale (3–9 range)^27^. The five p-values were adjusted for multiple testing by applying Benjamini–Hochberg correction.

### Risk factors for persistence of newly developed symptoms

A Generalised Estimating Equation (GEE) model was built (Table 2), with persistence of newly developed symptoms (binary variable), as the outcome, with the following independent variables: SARS-CoV-2 infection status, age, gender, number of comorbidities at baseline, recent vaccination and history of hospitalisation (Supplementary Table 4). As multiple visits were included in the model, the participant ID was considered the subject effect variable, with the exchangeable correlation structure to account for dependency between observations. Subgroup analysis, stratified by infection status, was conducted in a form of two additional GEE models: only in non-infected individuals and only in infected individuals (Supplementary Table 5).

**Table 2:** Risk factor analysis for persistent newly developed symptoms (GEE model). Presence/absence of persistent symptoms was considered as the binary outcome, with the subject effect incorporated in the model to account for dependence between observations. Visits 5–9 of CON-VINCE were considered, with the number of complete cases, n = 3,355 from 559 individuals. More details on the predictor variables used in the GEE model are provided in Supplementary Table 4.

Parameter	Odds ratio (OR)	Standard error (SE)	95% confidence interval (CI)	z-value	p-value
SARS-CoV-2 infection-No [reference]	1.000				
SARS-CoV-2 infection-Yes	**2.065**	0.225	[1.669; 2.556]	6.666	** < 0.001**
BRS score (resilience)	**0.581**	0.043	[0.503; 0.671]	-7.401	** < 0.001**
UCLA score (loneliness)	**1.185**	0.043	[1.104; 1.271]	4.703	** < 0.001**
Number of comorbidities	**1.152**	0.058	[1.044; 1.271]	2.822	**0.005**
Gender-Male [reference]	1.000				
Gender-Female	**1.346**	0.166	[1.057; 1.713]	2.413	**0.016**
Age	1.005	0.004	[0.997; 1.014]	1.213	0.225
Hospitalisation-No [reference]	1.000				
Hospitalisation-Yes	0.202	0.290	[0.012; 3.372]	-1.114	0.265
Recent vaccination-No [reference]	1.000				
Recent vaccination-Yes	0.853	0.168	[0.580; 1.255]	-0.806	0.420

Values that are statistically significant at 5% confidence interval are highlighted in bold.

### Odds ratios analysis

**Fig. 2:**
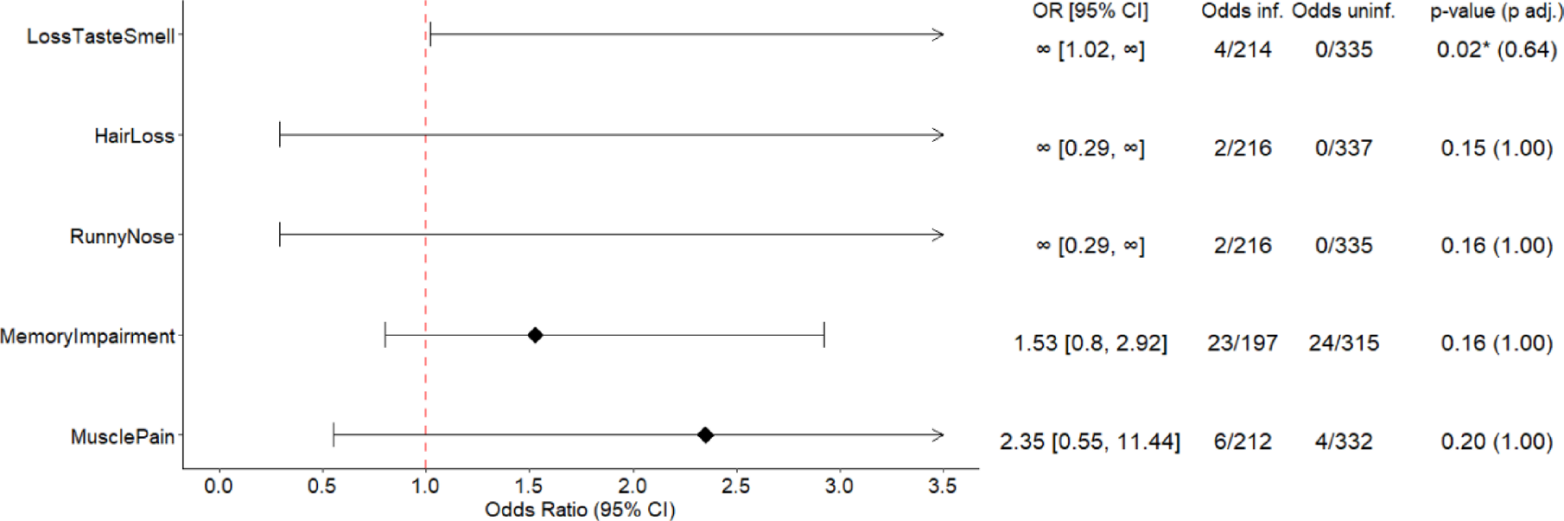
Forest plots. Odds ratios (ORs), odds and p-values of persistent symptoms between infected and non-infected participants at CON-VINCE visit 8. Only the odds ratios of the 5 symptoms with the 5 lowest p-values in the associated Fisher’s exact test are plotted. All SARS-CoV-2 infected and all non-infected participants with a known symptom status are presented. The odds represent the number of individuals with the persistent symptom divided by the number of individuals without the persistent symptom in the subgroup. The resulting ORs are presented in the forest plots. * Significantly different symptoms between the subgroups before adjustment for multiplicity at 5% confidence level.

**Fig. 3:**
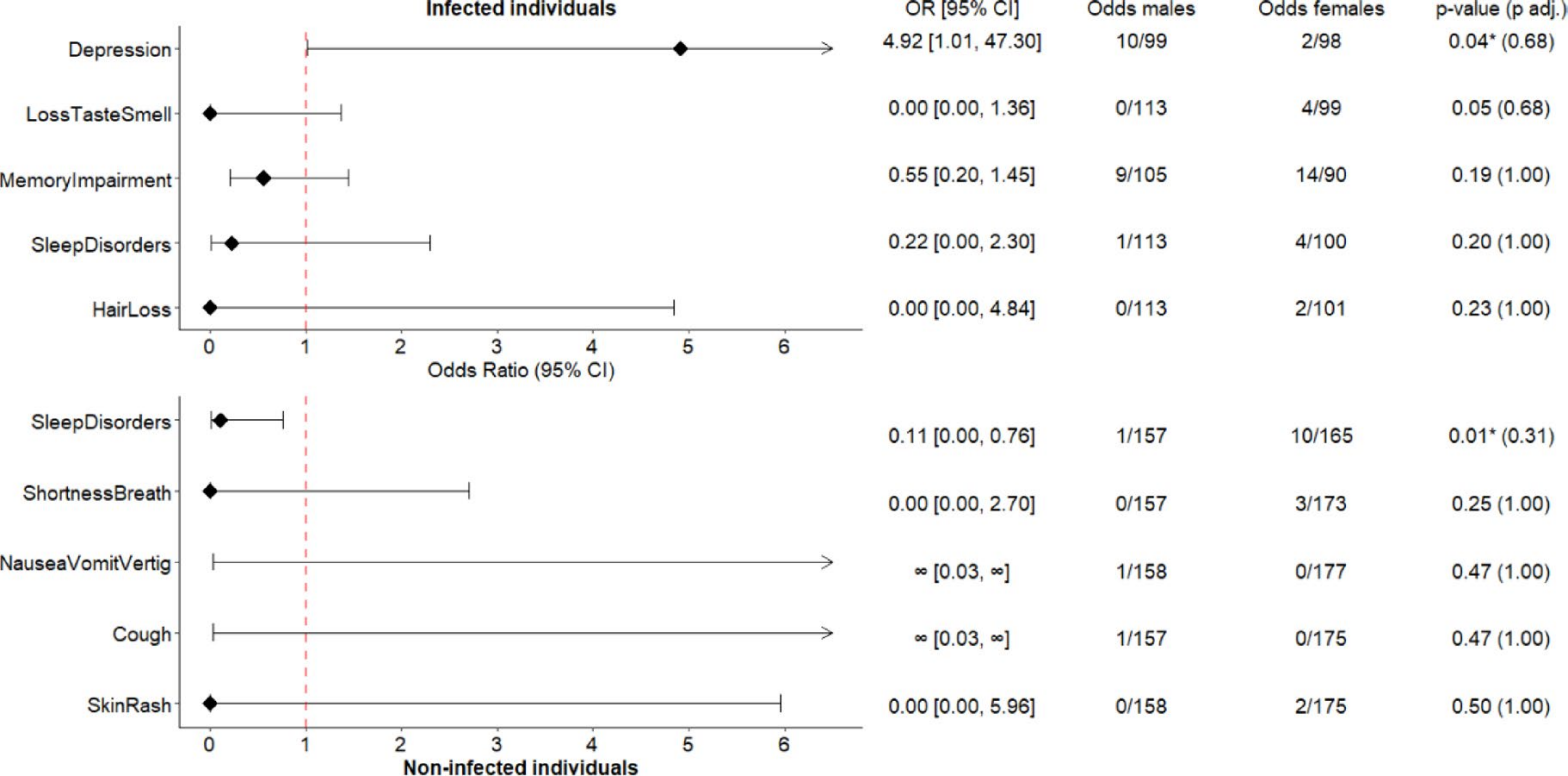
Forest plots. Odds ratios (ORs), odds and p-values of persistent symptoms between males and females, stratified by infection status at CON-VINCE visit 8. Only the odds ratios of the 5 symptoms with the 5 lowest p-values in the associated Fisher’s exact test within each group are plotted-infected at the top and non-infected at the bottom. The odds represent the number of males or females with the persistent symptom divided by the number of males or females without the persistent symptom, within the infected or non-infected subgroup. * Significantly different symptoms between the two genders before adjustment for multiplicity at 5% confidence level.

The difference in symptoms between non-infected and infected participants was assessed through odds ratios (ORs). The ORs, odds in each group, along with adjusted and unadjusted p-values from the Fisher’s exact tests are presented in Fig. 2, Supplementary Fig. 2, and Supplementary Table 6. The symptom difference between females and males was also assessed through the ORs (Fig. 3 and Supplementary Table 7). P-values were adjusted using Benjamini–Hochberg correction, adjusting for 27 parallel tests, and separately in males and separately in females. Forest plots were used to visualise the difference between the two considered groups, while describing the significance and certainty of the estimates through whiskers, representing the 95% confidence intervals (Figs. 2, 3).

Additionally, the comorbidities difference (at visit 0) between participants with persistent symptoms and those without persistent symptoms (at visit 8) was also assessed through ORs and forest plots (Fig. 5). Sensitivity analyses results, run on alternative study visits, are presented in Supplementary Table 8. Moreover, a subgroup analysis, stratified by infection status, was also conducted (Supplementary Table 9). P-values were adjusted using Benjamini–Hochberg correction (23 parallel comparisons).

### Clusters of symptoms and comorbidities

**Fig. 4:**
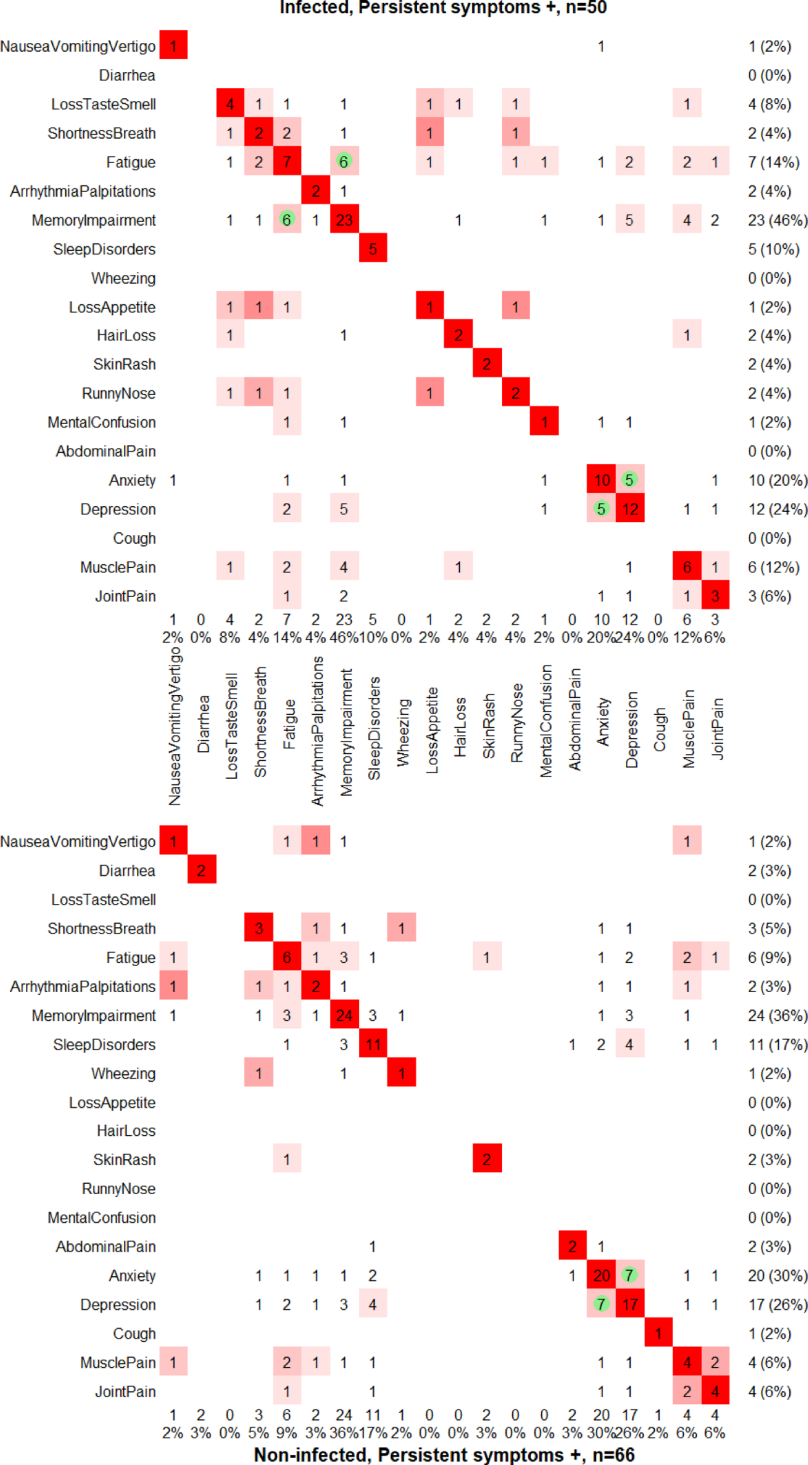
Pairwise combinations of persistent symptoms in SARS-CoV-2 infected individuals (top) and in non-infected individuals (bottom) at CON-VINCE visit 8. Only the symptoms present in at least one individual in any of the two groups (infected/non-infected) are plotted. Heat maps based on the Jaccard similarity index between symptoms. The Jaccard index ranges from zero for dissimilar (white) to one for similar (dark red), and it is not defined if both symptoms never occur. Significant at 5% level, FDR-adjusted p-values from a Fisher’s exact test are indicated by a green circle. The values on the right and at the bottom indicate the number of individuals with the given symptom, with the proportion of individuals with this symptom in the considered subgroup in brackets.

Assessment of symptoms combinations separately in infected and in non-infected was done through the Jaccard’s similarity index, and the Fisher’s exact tests (Fig. 4). The Jaccard index represents the number of individuals with both symptoms from a combination, divided by the number of individuals with at least one of the two symptoms. The heat maps for comorbidities were produced with the same methodology (Supplementary Fig. 3). All the p-values were adjusted using Benjamini–Hochberg correction (symptoms – 27 parallel tests; comorbidities – 23 parallel tests).

### Longitudinal analysis of anxiety and depression patterns

Only individuals with the three time points: visits 7, 8, 9 of CON-VINCE were included in the analysis. The longitudinal pattern consists of three time points (visits), annotated with either 0 or 1, where 0 indicates no anxiety/depression at a given visit, and 1 indicates anxiety/depression present at the visit, e.g. “001” or “101”. The proportions of the different longitudinal patterns were compared between infected and non-infected through Fisher’s exact tests (Supplementary Fig. 4). The difference in severity of depression and anxiety was assessed by comparing the CES-D and GAD-7 scores between the groups (mean ranks) with Mann–Whitney tests.

## Results

### Demographic characteristics

Demographic characteristics of the whole cohort at visit 8 of CON-VINCE, as well as of different subgroups, stratified by infection status and by post COVID status, are provided in Table 1. Persistent symptoms can be observed in both SARS-CoV-2 infected (post COVID) and uninfected groups (post COVID mimics).

At visit 8, 559 individuals had a known post COVID and infection status. The distribution between the two prevalent genders was balanced in the cohort (50.4% females and 48.8% males), while the mean age was 54.2 years. The post COVID group consisted of n = 50 individuals (those with persistent symptoms), corresponding to 22.7% of all infected individuals (50/220). The post COVID mimics group constituted n = 66 participants (19.5% individuals out of 339 non-infected individuals had persistent symptoms). The proportion of females was higher both in the post COVID and in post COVID mimics subgroups – 58.0% and 59.1% respectively, with no significant difference between the two groups (p-value = 1.000, adjusted p-value = 1.000). The mean (SD) age within these two subgroups was similar: 50.4 (14.5) in post COVID group and 54.1 (14.0) in post COVID mimics group (p-value = 0.165, adjusted p-value = 0.661). Furthermore, 40% (n = 20) of infected participants with persistent symptoms at visit 8 did not report any comorbidity at baseline. However, 27.3% (n = 18) of non-infected participants did not have any comorbidities at baseline, yet presented persistent post COVID symptoms at visit 8. The proportion of comorbid individuals was not significantly different between post COVID and post COVID mimics groups (p-value = 0.166, adjusted p-value = 0.661).

We further investigated whether individuals’ ability to adapt, and recover from stressful situations, such as the pandemic and the imposed health policy measures, could explain why non-infected individuals may have developed new persistent symptoms. The results of comparison of the resilience (BRS score, median [Q1, Q3]) in post COVID (3.7 [3.0, 4.0]) and in post COVID mimics (3.2 [2.7, 3.7]) suggest that infected individuals were more resilient than non-infected ones, yet, not after adjusting for multiplicity (p-value = 0.010, adjusted p-value = 0.052). The loneliness UCLA scale scores were similar between post COVID and post COVID mimics (p-value = 0.979, adjusted p-value = 1.000).

### Frequency of post COVID/post COVID mimics at different study visits

Evolution of the distribution of participants by infection status and by presence of persistent symptoms at different study visits is presented in Fig. 1 and Supplementary Table 2. Both infected and uninfected participants reported persistent symptoms over the course of the study. Moreover, the exact proportions of infected and non-infected participants, as well as the proportions of participants with and without persistent symptoms varied from visit to visit.

### Risk factors for presenting newly developed persistent symptoms

We further investigated which risk factors are associated with presenting persistent symptoms using a Generalised Estimating Equations (GEE) model, n = 3,355 observations (from 559 individuals). SARS-CoV-2 infection was one of the most impactful risk factor for development of persistent symptoms (OR: 2.07, 95% CI: [1.67, 2.56]), when adjusting for age, gender, recent vaccination, BRS score (resilience), UCLA score (loneliness), number of comorbidities and history of hospitalisation (Table 2). Additionally, females had significantly higher odds of reporting persisting symptoms (OR: 1.35, 95% CI: [1.06, 1.71]) when compared to males. Furthermore, having more comorbidities significantly increases the odds of reporting persistent symptoms, with an OR = 1.15 (95% CI: [1.04, 1.27]) for each additional comorbidity present at baseline. Higher resilience was associated with lower odds of persistent symptoms-OR = 0.58 (95% CI: [0.50, 0.67]) for each additional point on the BRS scale. People with greater loneliness were also more prone to presenting persistent symptoms-OR: 1.19 (95% CI: [1.10, 1.27]) per point on the UCLA short scale. Recent vaccination, age and hospitalisation history had no effect on the odds of reporting persistent newly developed symptoms.

The full results of the analysis conducted separately in each of the two subgroups (infected, n = 919 questionnaire instances; and non-infected, n = 2,453 questionnaire instances) are provided in Supplementary Table 5. In the subgroup of infected participants, being a female increased the odds of experiencing persistent symptoms (OR: 1.78, 95% CI: [1.21; 2.61], p-value = 0.003), whereas this effect was not observed in the subgroup of non-infected individuals (OR: 1.17, 95% CI: [0.88; 1.55], p-value = 0.285). On the contrary, the number of comorbidities significantly increased the odds of persistent symptoms in the subset of non-infected individuals by 15% (OR: 1.15, 95% CI: [1.02; 1.29], p-value = 0.019), but not in the infected subgroup (OR: 1.12, 95% CI: [0.94; 1.34], p-value = 0.22). The effects of resilience and loneliness were significant, and of similar size and direction in both subgroups, similar to the main analysis findings (Supplementary Table 5).

### Difference in symptoms of infected and non-infected individuals

To better understand if there is a difference between the persistence of symptoms reported by infected and non-infected participants, the odds of each symptom were assessed. A visual representation of the odds in the two groups is provided in Fig. 2 (Supplementary Fig. 2). A sensitivity analysis for different study visits can be found in Supplementary Table 6.

Loss of taste and/or smell was more frequent in the SARS-CoV-2 infected group than in the non-infected group, prior to adjusting for multiplicity (p-value = 0.024, adjusted p-value = 0.641, n = 553).

A detailed overview of symptoms’ comparisons between SARS-CoV-2 infected and non-infected individuals is provided in Supplementary Table 6 and Supplementary Fig. 2.

### Difference in symptoms of males and females, stratified by infection status

We further investigated whether there are any differences in specific symptoms between females and males stratified by infection status (Fig. 3 and in Supplementary Table 7).

Infected males experienced persistent depression more frequently than infected females, before adjusting for multiplicity (OR = 4.95 [1.01, 47.30]; p-value = 0.035, adjusted p-value = 0.677, n = 209).

Non-infected females reported persistent sleep disorders more often than non-infected males, yet, not significantly after multiplicity adjustment (OR = 0.10 [0.00, 0.76]; p-value = 0.012; adjusted p-value = 0.311, n = 333).

### Symptom clusters, stratified by infection status

Pairwise associations of symptoms were calculated in the infected and non-infected subgroups to identify potential co-occurring symptoms in each subgroup (Fig. 4).

Two combinations of persistent symptoms occurring in SARS-CoV-2 infected individuals (n = 50) were identified: (1) fatigue and memory impairment, (2) depression and anxiety, while in non-infected (n = 66) individuals depression and anxiety was the only pair of significantly associated symptoms (see Supplementary Table 3 for an exploratory cluster analysis of the co-occurrence of comorbidities with symptoms).

Interestingly, the combination of anxiety and depression was the most frequently reported in both groups, suggesting an infection-unrelated occurrence of these. Moreover, there was no difference in the dynamics of presence of depression or anxiety between infected and non-infected participants (n = 566). These conditions persisted or resolved in similar patterns, independently of infection status (Supplementary Fig. 4).

We further investigated whether infected individuals with depression (n = 12) or anxiety (n = 10) experience these symptoms with higher severity when compared to non-infected individuals with depression (n = 17) or anxiety (n = 20), and found no significant differences in the severity scores of depression (CES-D) and anxiety (GAD-7) between infected and non-infected individuals (p-value = 0.98 and 0.91, respectively). The median score of depression (CES-D) was 23 [Q1, Q3: 18, 30] in infected depressed individuals, and 22 [Q1, Q3: 19, 25] in non-infected depressed individuals at visit 8. While the median anxiety score (GAD-7) was 8 [Q1, Q3: 6, 11] in infected individuals with anxiety, and 8 [Q1, Q3: 6, 9] in the non-infected group with anxiety at visit 8.

### Associations between comorbidities and symptoms

A deeper analysis of the comorbidities was conducted to better understand which comorbidities contribute more to the reporting of newly developed persistent symptoms.

**Fig. 5:**
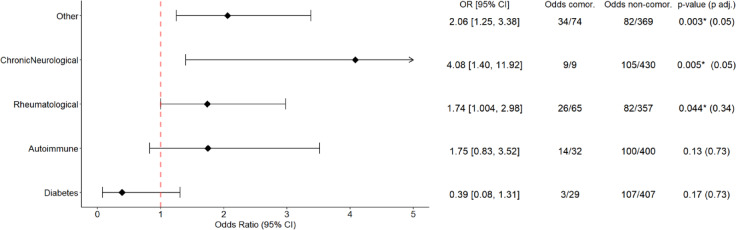
Forest plots. Odds ratios (ORs), odds and p-values of presenting any persistent symptoms at CON-VINCE visit 8 between comorbid and non-comorbid participants at baseline visit 0. Only the odds ratios of the 5 comorbidities with the 5 lowest p-values in the associated Fisher’s exact test are plotted. The values represent the number of individuals with any persistent symptom divided by the number of individuals without any persistent symptom at visit 8 in the comorbid or non-comorbid subgroups. * Significantly different comorbidities between the subgroups before adjustment for multiplicity at 5% confidence level.

The odds of presenting any persistent symptoms at the CON-VINCE visit 8, given the presence of a comorbidity at baseline, reveals that chronic neurological or rheumatological disorders are associated with an increased risk of persistent symptoms at the whole cohort level, before adjusting for multiplicity (Fig. 5; n = 553 and n = 530 included in comparisons). Please refer to Supplementary Table 8 for the full results and the comparison with other study visits. When stratifying the study population by infection status, non-infected individuals with rheumatological or neurological disorders had higher frequency of symptoms than non-infected individuals without these comorbidities, before adjusting for multiplicity (Supplementary Table 9; n = 320 and n = 333 included in comparisons). Furthermore, no statistically significant co-occurrence of comorbidities was identified in the subgroup of post COVID (n = 50) and post COVID-mimics (n = 66) at visit 8 (Supplementary Fig. 3).

These findings suggest that individuals with chronic conditions have a higher likelihood of suffering from persistent symptoms, especially in non SARS-CoV-2 infected participants.

## Discussion

Our study provides a robust assessment of the risk factors and incidence of post COVID and post COVID mimics, based on a national cohort of the Luxembourgish population over a 4 year follow-up and provides evidence to better understand the complex relationship between the COVID-19 pandemic and the development of new persisting symptoms, irrespective of a SARS-CoV-2 infection.

In our study, the proportion of individuals who developed persistent symptoms was 22.7% in the infected group and 19.5% in the non-infected group, resulting in an absolute difference of 3.2%. In other studies, such as in the Lifelines cohort^29^, it was demonstrated that the proportion of participants developing post COVID symptoms was 21.4% in the infected group, and 8.7% in the matched non-infected controls. Whereas very similar percentages were observed for infected individuals (22.7% in our study vs 21.4% in Lifelines), more post COVID mimics were identified in our study, compared to Lifelines (19.5% vs 8.7%). The difference between those findings may partially be explained by the inclusion of newly developed depression/anxiety in the definition of post COVID. Including these two symptoms may have inflated the number of post COVID mimics, as 45.5% of post COVID mimics developed at least one of these two symptoms, when compared to the post COVID group (34.0%).

Our results confirm findings of previous studies regarding risk factors for post COVID, with female gender (OR: 1.78, 95% CI: [1.21; 2.61]) being associated with increased likelihood of persistent symptoms in infected individuals^30^. At the same time, lower resilience scores (OR: 0.58, 95% CI: [0.50; 0.67]) were associated with higher odds of persistent symptoms in both infected^30^ and non-infected groups. This suggests that individual vulnerability factors play a crucial role in the development of persistent symptoms, whether related to infection or not. Higher loneliness scores were also associated with the higher chance of persistent symptoms, in both infected and non-infected individuals (OR: 1.19, 95% CI: [1.10, 1.27] per 1 point on the UCLA 3-item scale). This suggests that loneliness on the one hand may increase the likelihood of persistent symptoms, and on the other hand may be a consequence of the pandemic^31^. Interestingly, we found that presence of comorbidities only played a significant role in non-infected participants (OR: 1.15, 95% CI: [1.02, 1.29] per comorbidity), suggesting that the effect of the pandemic was more pronounced in comorbid non-infected individuals.

Moreover, we confirm that SARS-CoV-2 infection itself increases the risk to present with persistent symptoms (OR: 2.07, 95% CI: [1.67; 2.56]), but, most importantly, also demonstrate that similar symptoms also occur in non-infected individuals such as depression and anxiety. Interestingly, the findings of a recent study indicated that there were no statistically significant differences in individual symptom characteristics between long COVID and long COVID mimics^32^. This highlights the need to carefully differentiate between symptoms specifically related to the infection, and the ones caused by secondary processes (not infection-related).

Furthermore, we demonstrate that gender strongly correlates with overall mental health burden, which might be due to the pandemic, with females more frequently reporting fatigue (exhaustion) and depression^33^. The higher prevalence, longer persistence and severity of symptoms in women has been consistently reported in several other studies^12,29^ and mirrors patterns seen in other post-viral and autoimmune conditions^34,35^.

An analysis of individual symptoms and symptom clusters revealed additional patterns, e.g. loss of taste or smell is more likely to be self-reported in infected individuals than in uninfected controls (reached significance only prior to multiple testing adjustment)^36^. Importantly, we identified that there exists a significant combination of memory impairment and fatigue that occurs specifically in infected individuals (86% individuals with fatigue also have memory impairment, while in non-infected individuals only 50% had both symptoms). A similar “chronic fatigue like”-symptom cluster has been previously identified by Gentilotti et al.^37^.

While infection-specific symptom combinations were identified, a cluster of anxiety and depression was identified regardless of the infection status—42% and 41% individuals with depression also had anxiety, in infected and non-infected groups respectively. In our study, we did not find an increase in newly developed depression/anxiety symptoms in individuals after confirmed SARS-CoV-2 infection when compared to non-infected controls. Interestingly, the severity of depression and anxiety in individuals with those symptoms was similar in infected and non-infected groups, according to the respective severity scores (CES-D and GAD-7). Additionally, trajectory analysis of anxiety and depression symptoms, showed similar patterns over time in both infected and non-infected groups, again emphasising that these symptoms may rather be a side effect of social isolation in times of lockdown during the pandemic, as well as a lower individual resilience. This is important, as other studies have proposed an interaction of SARS-CoV-2 infection inducing e.g. depression/anxiety symptoms^38^, and part of the observed increase might be rather due to the impact of the pandemic and potentially the implemented public health measures^18,19^. Lastly, co-occurrence of these two symptoms is also known to occur in the general population independently of the pandemic^39^. This suggests that examining symptom patterns, rather than individual symptoms, may be more valuable in distinguishing post COVID from pandemic-related effects. Overall, this has important implications, highlighting the need to consider pandemic-related stressors alongside SARS-CoV-2 infection status when evaluating persistent symptoms, and not SARS-CoV-2 infection exclusively.

Furthermore, the role of comorbidities in increasing the risk for persistent symptoms in non-infected individuals provides important context for understanding individual vulnerability. A meta-analysis based on 41 studies has shown that specific comorbidities i.e. diabetes, immunosuppression, asthma, among others are associated with post COVID^40^, however this meta-analysis showed multiple limitations such as the inclusion of studies with heterogeneous diagnosis criteria (i.e. self-reported vs clinician-reported vs RT-PCR confirmed test vs antigen test), and different definition of symptoms among the studies. On the one hand, our results show that rheumatological and chronic neurological disorders are associated with higher likelihood of presenting persistent symptoms independently of SARS-CoV-2 infection (before adjusting for multiplicity). Rheumatological and chronic neurological disorders are complex diseases that can present with persistent, sometimes overlapping symptoms characterised by fatigue, pain and cognitive dysfunction, among others^41,42^, demonstrating how important it is to exclude pre-existing health conditions that may explain the persistence of post COVID-like symptoms. On the other hand, individuals not reporting any comorbidity at baseline also developed new persistent symptoms, independent of the infection status. Pre-existing conditions or individual factors such as the individual’s resilience to adverse situations or social isolation may create a susceptibility to persistent symptoms through mechanisms that are not necessarily specific to SARS-CoV-2 infection. We therefore showed that higher loneliness and lower resilience are risk factors for the development of persistent symptoms, independently of the infection status. Previous studies have shown that these risk factors are predictive of worse mental and physical health^43–45^. Moreover, resilience and/or social contact played a crucial role in maintaining mental health during the pandemic, and may act as protective factors against symptoms of depression^46^, anxiety^44,47^, or stress^48^.

Finally, our results regarding persistent symptoms after SARS-CoV-2 infection must be interpreted in the context of the prevalence of symptoms in the analysed sample. Although we confirm that SARS-CoV-2 infection is associated with a greater likelihood of developing persistent symptoms (OR: 2.07, 95% CI: [1.67; 2.56]), careful differentiation between infection-specific and general symptoms is crucial for accurate characterisation of the post COVID-19 condition. Moreover, we show that choosing a different study visit may result in slightly different significant odds ratios of symptoms and comorbidities, which may be explained by the direct and indirect impact of covariates on the outcome (post COVID), such as the number of months between the infection and the time of assessment^49^, the prevalent viral variant^50^, and the proportion of infected to non-infected individuals included in the analyses.

## Limitations

The CON-VINCE study captured detailed questionnaire information on symptoms, pre-existing medical conditions and mental health, but – due to the pandemic conditions – did not include an in-person visit for clinical assessments of participants’ persistent symptoms. A clear definition of post COVID syndrome was therefore not possible for the study participants. Thus, we have applied the stringent WHO post COVID definition and implemented a range of criteria to conclude that an individual had post COVID and that the symptoms are truly newly developed and persistent. Importantly, only new symptoms that were not yet present at baseline were considered. However, the “memory impairment” symptom was an exception, as the question was first asked at CON-VINCE visit 7, so it was not possible to adjust for it, using the previous CON-VINCE visits. For this specific symptom it is not possible to conclude with confidence whether it was newly developed or not, even though it was persistent. Thus, this might have resulted in an overestimation of frequency of newly developed memory impairment, nevertheless, this symptom co-occurred more frequently with fatigue in a “chronic fatigue like”-cluster only in infected participants.

Furthermore, other symptoms such as arrhythmia/palpitations, sleep disorders, loss of appetite, and hair loss were not fully adjusted for their presence at the baseline visit since this information started to be collected later on in the study, as indicated in the Methods section.

## Conclusion

Our study identifies specific symptom patterns that differentiate between post COVID (as defined by SARS-CoV-2 infection) and post COVID mimics in a group of individuals followed over 4 years. Here, the combination of fatigue and memory impairment appeared specifically more often in infected individuals, while the anxiety and depression cluster was observed regardless of infection status, highlighting that these symptoms also need to be considered outside of the context of post COVID syndrome and causes other than SARS-CoV-2 infection need to be examined. Yet, we could not verify with certainty that memory impairment was indeed a new symptom developed over the course of this study, as this symptom was not collected at the baseline visit. Importantly, we describe a chronic fatigue-like cluster of symptoms only in individuals with prior SARS-CoV-2 infection that is not observed in uninfected individuals. Establishing a clinically defined post COVID cohort alongside a control-matched population and detailed biological biomarkers is crucial to further advance in post COVID screening and improvement of management strategies of affected individuals.

## Supplementary Information

Below is the link to the electronic supplementary material.

Supplementary Material 1

## Data Availability

The dataset for this manuscript is not publicly available as it is linked to the CON-VINCE and ORCHESTRA Luxembourg studies and their internal regulations. Any requests for accessing the dataset can be directed to orchestra.luxembourg@lih.lu. All data of the manuscript will be provided upon reasonable request and approval by the study executive committee.

## Code availability

The code used to conduct the analysis presented in this manuscript is accessible at https://gitlab.lcsb.uni.lu/DMG/long-term-effects-of-sars-cov-2-infection-and-covid-19-pandemic.
