# Supplementary material for: Co-occurrence of memory impairment and fatigue distinguishes post COVID from pandemic-related health effects in the 4-year CON-VINCE cohort study
Source: Sci Rep. 2025 Oct 27;15:37381. doi: 10.1038/s41598-025-19984-7 (PMC12559301; doi:10.1038/s41598-025-19984-7)
Supplement: Supplementary file 1 — Supplementary Material 1 [file 41598_2025_19984_MOESM1_ESM.pdf]

Co-occurrence of memory impairment and fatigue distinguishes post COVID from pandemic-related health effects in the 4-year CON-VINCE cohort study

*Patricia Martins Conde *†[1], Dmitry Bulaev†[2], Armin Rauschenberger[1,2], Jochen Ohnmacht[2], Joëlle V. Fritz[2], Marc P. O'Sullivan[2,3], François Ancien[1], Soumyabrata Ghosh[1], Olena Tsurkalenko[2,4], Alexey Kolodkin[2], Venkata Satagopam[1], Michel Vaillant[2], Jochen Klucken[1,4], Rejko Krüger[1,2,4], CON-VINCE/ORCHESTRA study consortium*

†*Joint first authorship*

*Corresponding author – Patricia Martins Conde (patricia.martinsconde@uni.lu)

[1] Luxembourg Centre for Systems Biomedicine (LCSB), University of Luxembourg, Esch-sur-Alzette, Luxembourg

[2] Luxembourg Institute of Health, Strassen, Luxembourg

[3] Eurostat, European Commission, Luxembourg, Luxembourg

[4] Centre Hospitalier de Luxembourg, Luxembourg, Luxembourg

Supplementary information

Supplementary tables:

- **Supplementary table 1**. List of all self-reported symptoms and comorbidities by CON-VINCE participants.
- **Supplementary table 2.** Presence of persistent symptom at different visits of the study, stratified by SARS-CoV-2 infection status.
- **Supplementary table 3.** Identified clusters of comorbidities and symptoms present in individuals reporting persistent symptoms, stratified by SARS-CoV-2 infection status at CON-VINCE visit 8.
- **Supplementary table 4**. Description of predictor variables used in the risk factor analysis. Generalised Estimating Equations model with binary outcome - presence/absence of persistent newly developed symptoms.
- **Supplementary table 5.** Subgroup risk factor analysis, only in infected and in only in non-infected individuals. Results of Generalised Estimating Equations (GEE) models.
- **Supplementary table 6.** Odds, odds ratios with confidence intervals, adjusted and unadjusted Fisher's test p-values for individual symptom comparisons between SARS-CoV-2 infected and non-infected individuals at the different study visits.
- **Supplementary table 7.** Odds, odds ratios with confidence intervals, adjusted and unadjusted Fisher's test p-values for individual symptom comparisons between SARS-CoV-2 males and females in infected and in non-infected groups at CON-VINCE visit 8.
- **Supplementary table 8.** Odds, odds ratios with confidence intervals, adjusted and unadjusted Fisher's test p-values for individual comorbidities comparisons between individuals with and without persistent symptoms at the different study visits.
- **Supplementary table 9.** Odds, odds ratios with confidence intervals, adjusted and unadjusted Fisher's test p-values for individual comorbidities comparisons between SARS-CoV-2 infected and non-infected individuals at CON-VINCE visit 8.

Supplementary figures:

- **Supplementary figure 1.** CON-VINCE / ORCHESTRA Europe study visits distribution of dates.
- **Supplementary figure 2.** Odds of 27 symptoms in all SARS-CoV-2 infected and non-infected participants at CON-VINCE visit 8.
- **Supplementary figure 3.** Pairwise combinations of comorbidities in SARS-CoV-2 infected individuals (top) and in non-infected individuals (bottom) reporting at least one persistent symptom at CON-VINCE visit 8.
- **Supplementary figure 4.** Longitudinal trajectories of depression and anxiety, stratified by SARS-CoV-2 infection status.

Supplementary table 1. List of all self-reported symptoms and comorbidities by CON-VINCE participants.

Symptoms	Comorbidities
Nausea / Vomiting / Vertigo	Chronic pulmonary disease
Fever	Diabetes
Headache	Chronic kidney disease
Diarrhoea	Rheumatological disorder
Loss of taste and/or smell	Dementia
Shortness of breath	Stroke
Fatigue	Mild liver disease
Arrhythmia / Palpitations	Severe liver disease
Chest pain	Malnutrition
Memory impairment	Chronic neurological disorder
Sleep disorders	Neurodegenerative disease
Wheezing	Cancer
Ear pain	Chronic haematological disease
Lymph nodes	HIV
Loss appetite	Autoimmune disease
Hair loss	Organ transplantation
Skin rash	Immunosuppression
Runny nose	Psychiatric disease
Epilepsy	Chronic cardiac disease
Sore throat	Non-chronic cardiovascular disease
Mental confusion	Hypertension
Abdominal pain	Asthma
Anxiety	Other chronic diseases
Depression	
Cough	
Muscle pain	
Joint pain	

Supplementary table 2. Presence of persistent symptom at different visits of the study, stratified by SARS-CoV-2 infection status.

	Infection	Persistent symptoms	Visit 5	Visit 6	Visit 7	Visit 8	Visit 9
**Post COVID** Infected, Persistent symptoms +	+	+	13 (12.5%)	9 (31.0%)	30 (22.4%)	50 (22.7%)	129 (28.1%)
Infected, Persistent symptoms -	+	-	91 (87.5%)	20 (69.0%)	104 (77.6%)	170 (77.3%)	330 (71.9%)
**Post COVID MIMICS** Non-infected, Persistent symptoms +	-	+	119 (11.6%)	71 (23.7%)	113 (15.4%)	66 (19.5%)	35 (26.5%)
Non-infected, Persistent symptoms -	-	-	906 (88.4%)	229 (76.3%)	619 (84.6%)	273 (80.5%)	97 (73.5%)

Supplementary table 3. Identified clusters of comorbidities and symptoms present in individuals reporting persistent symptoms, stratified by SARS-CoV-2 infection status at CON-VINCE visit 8.

**CON-VINCE visit 8, adjusted for presence of symptoms at baseline**			
**Infected, Persistent symptoms +** (post COVID)		**Non-infected, Persistent symptoms +** (post COVID MIMICS)	
**Pair of Symptoms / Comorbidities in cluster**	**Presence**	**Pair of Symptoms / Comorbidities in cluster**	**Presence**
Fatigue & Memory impairment	12% (6/50)	Anxiety & Depression	10.6% (7/66)
Anxiety & Depression	10% (5/50)	Memory impairment & Hypertension	10.6% (7/66)
Memory impairment & Depression	10% (5/50)	Anxiety & Hypertension	9.1% (6/66)
Memory impairment & hypertension	10% (5/50)	Memory impairment & Rheumatological disorder	9.1% (6/66)
Memory impairment & Muscle pain	8% (4/50)	Anxiety & Rheumatological disorder	9.1% (6/66)
Memory impairment & Rheumatological disorder	8% (4/50)	Chronic neurological disorder & Hypertension	7.6% (5/66)
Non-chronic cardiac disease & Hypertension	8% (4/50)	Rheumatological disorder & Hypertension	7.6% (5/66)
Memory impairment & Psychiatric disease	6% (3/50)	Sleep disorders & Rheumatological disorder	7.6% (5/66)
Asthma & Hypertension	6% (3/50)	Rheumatological disorder & Autoimmune disease	7.6% (5/66)
Memory impairment & Non-chronic cardiac disease & Hypertension	6% (3/50)		

Supplementary table 4. Description of predictor variables used in the risk factor analysis. Generalised Estimating Equations model with binary outcome - presence/absence of persistent newly developed symptoms.

Independent variable	Description
SARS-CoV-2 infection - Yes	Infection identified as described in the "Infection status definition" section
Gender - Female	Gender "Female" at birth. As the number of individuals with diverse/unknown gender was very low, these individuals were not included in the risk factor analysis.
BRS scale score (resilience)	Score obtained from the 6-item Brief Resilience scale, ranging from 1 to 5.<br>Derived as the sum of 6 items (with a range from 1 to 5), divided by 6.
UCLA scale score (loneliness)	Score obtained from the 3-item short version UCLA scale, ranging from 1 to 3.<br>Derived as the sum of 3 items, with a resulting range from 3 to 9.
Number of comorbidities	The count of comorbidities (0-23)
Recent vaccination - Yes	Received a SARS-CoV-2 vaccine 0-7 days before the day of questionnaire completion
Hospitalisation - Yes	Have been hospitalised for COVID-19
Age	Age in years at the day of questionnaire completion

Supplementary table 5. Subgroup risk factor analysis, only in infected and in only in non-infected individuals. Results of Generalised Estimating Equations (GEE) models.

	SARS-CoV-2 infected only					SARS-CoV-2 non-infected only				
Risk factor	Odds Ratio (OR)	Standard error (SE)	95% Confidence interval (CI)	z-value	p-value	Odds Ratio (OR)	Standard error (SE)	95% Confidence interval (CI)	z-value	p-value
**BRS scale score (higher score - more resilience)**	**0.562**	**0.064**	**[0.449; 0.703]**	**-5.044**	**<0.001**	**0.569**	**0.051**	**[0.477; 0.678]**	**-6.308**	**<0.001**
**Gender - Male [reference]**	**1.000**									
**Gender - Female**	**1.776**	**0.349**	**[1.208; 2.610]**	**2.922**	**0.003**	1.168	0.170	[0.879; 1.552]	1.069	0.285
**UCLA scale score (higher score - higher loneliness)**	**1.200**	**0.083**	**[1.047; 1.374]**	**2.629**	**0.009**	**1.190**	**0.051**	**[1.094; 1.296]**	**4.037**	**<0.001**
**Number of comorbidities**	1.120	0.103	[0.936; 1.340]	1.234	0.217	**1.149**	**0.068**	**[1.024; 1.29]**	**2.354**	**0.019**
Age	1.008	0.007	[0.994; 1.023]	1.141	0.254	1.006	0.005	[0.996; 1.016]	1.132	0.258
Recent vaccination - No [reference]	1.000									
Recent vaccination - Yes	0.802	0.441	[0.273; 2.356]	-0.401	0.688	0.837	0.185	[0.544; 1.29]	-0.805	0.421

Supplementary table 6. Odds, odds ratios with confidence intervals, adjusted and unadjusted Fisher's test p-values for individual symptom comparisons between SARS-CoV-2 infected and non-infected individuals at the different study visits. CON-VINCE visit 8 is the main visit. Abbreviations: S+ = with persistent newly developed symptoms; S- = without persistent symptoms; I+ = SARS-CoV-2 infected. I- = not infected by SARS-CoV-2. OR = odds ratio; 95% CI = confidence interval 95%. * Indicates significant p-values at 5% confidence level before Benjamini-Hochberg multiple testing adjustment; *** significant after Benjamini-Hochberg multiplicity adjustment.

	**CON-VINCE V8 [MAIN ANALYSIS]**				CON-VINCE V7				CON-VINCE V9				CON-VINCE V8 UNADJUSTED FOR SYMPTOMS AT BASELINE			
Symptom	I+ S+ / I+ S-	I- S+ / I- S-	OR [95% CI]	Adjusted p-value [p-value]	I+ S+ / I+ S-	I- S+ / I- S-	OR [95% CI]	Adjusted p-value [p-value]	I+ S+ / I+ S-	I- S+ / I- S-	OR [95% CI]	Adjusted p-value [p-value]	I+ S+ / I+ S-	I- S+ / I- S-	OR [95% CI]	Adjusted p-value [p-value]
**Loss of taste / smell**	4/214	0/335	∞ [1.02, ∞]	**0.641 [0.024*]**	3/129	0/727	∞ [2.3, ∞]	**0.096 [0.004*]**	5/448	0/130	∞ [0.26, ∞]	1 [0.592]	12/233	3/353	6.04 [1.61, 33.69]	**0.033*** [0.002*]**
Hair loss	2/216	0/337	∞ [0.29, ∞]	1 [0.154]	1/133	4/723	1.36 [0.03, 13.87]	1 [0.572]	5/447	2/128	0.72 [0.12, 7.61]	1 [0.656]	12/231	11/346	1.63 [0.65, 4.16]	1 [0.281]
Runny nose	2/216	0/335	∞ [0.29, ∞]	1 [0.155]	1/132	0/732	∞ [0.14, ∞]	0.86 [0.154]	2/452	0/131	∞ [0.05, ∞]	1 [1]	9/235	13/342	1.01 [0.37, 2.6]	1 [1]
**Memory impairment**	23/197	24/315	1.53 [0.8, 2.92]	1 [0.164]	0/133	0/731	0 [0, ∞]	1 [1]	62/397	10/122	1.9 [0.93, 4.29]	0.955 [0.071]	53/192	43/316	2.03 [1.27, 3.23]	**0.033*** [0.002*]**
**Muscle pain**	6/212	4/332	2.35 [0.55, 11.44]	1 [0.202]	3/130	13/714	1.27 [0.23, 4.7]	1 [0.725]	9/442	8/124	0.32 [0.11, 0.96]	**0.893 [0.033*]**	18/225	33/323	0.78 [0.41, 1.47]	1 [0.459]
**Fatigue**	7/211	6/330	1.82 [0.52, 6.66]	1 [0.389]	6/127	11/712	3.05 [0.91, 9.2]	**0.482 [0.036*]**	15/440	3/128	1.45 [0.4, 7.96]	1 [0.775]	17/227	22/334	1.14 [0.55, 2.3]	1 [0.738]
Loss of appetite	1/217	0/335	∞ [0.04, ∞]	1 [0.394]	0/132	1/726	0 [0, 214.08]	1 [1]	1/452	0/130	∞ [0.01, ∞]	1 [1]	3/242	1/355	4.39 [0.35, 231.38]	1 [0.31]
Mental confusion	1/218	0/336	∞ [0.04, ∞]	1 [0.395]	1/132	1/727	5.49 [0.07, 431.32]	0.86 [0.285]	0/453	0/132	0 [0, ∞]	1 [1]	2/243	0/356	∞ [0.27, ∞]	0.895 [0.166]

Diarrhoea	0/220	2/336	0 [0, 8.18]	1 [0.522]	0/133	0/727	0 [0, ∞]	1 [1]	0/456	1/131	0 [0, 11.29]	1 [0.224]	3/242	7/351	0.62 [0.1, 2.76]	1 [0.747]
Abdominal pain	0/220	2/336	0 [0, 8.18]	1 [0.522]	0/133	0/727	0 [0, ∞]	1 [1]	1/455	0/132	∞ [0.01, ∞]	1 [1]	4/241	6/352	0.97 [0.2, 4.16]	1 [1]
Sleep disorders	5/215	11/324	0.69 [0.18, 2.18]	1 [0.608]	4/130	17/709	1.28 [0.31, 4.02]	1 [0.555]	15/440	4/128	1.09 [0.34, 4.59]	1 [1]	31/214	66/289	0.64 [0.39, 1.03]	0.5 [0.056]
Arrhythmia / palpitations	2/217	2/334	1.54 [0.11, 21.36]	1 [0.649]	1/132	1/722	5.45 [0.07, 428.36]	0.86 [0.287]	5/450	1/130	1.44 [0.16, 68.82]	1 [1]	6/238	11/346	0.79 [0.24, 2.38]	1 [0.804]
Skin rash	2/217	2/335	1.54 [0.11, 21.42]	1 [0.649]	1/131	6/723	0.92 [0.02, 7.68]	1 [1]	1/451	1/131	0.29 [0, 22.97]	1 [0.401]	5/239	10/347	0.73 [0.19, 2.37]	1 [0.608]
Anxiety	10/201	20/317	0.79 [0.32, 1.81]	1 [0.7]	10/122	51/668	1.07 [0.47, 2.21]	1 [0.854]	37/411	9/121	1.21 [0.55, 2.93]	1 [0.715]	69/170	91/266	1.19 [0.81, 1.74]	1 [0.396]
Depression	12/199	17/320	1.14 [0.48, 2.58]	1 [0.845]	5/127	48/671	0.55 [0.17, 1.42]	0.86 [0.244]	36/412	6/124	1.8 [0.73, 5.36]	1 [0.249]	45/194	70/287	0.95 [0.61, 1.47]	1 [0.833]
Nausea / vomiting / vertigo	1/219	1/337	1.54 [0.02, 121.03]	1 [1]	0/133	1/726	0 [0, 212.48]	1 [1]	1/455	1/131	0.29 [0, 22.77]	1 [0.399]	5/240	4/354	1.84 [0.39, 9.38]	1 [0.497]
Shortness breath	2/216	3/332	1.02 [0.09, 9.02]	1 [1]	2/129	3/727	3.75 [0.31, 33.04]	0.86 [0.168]	5/448	1/131	1.46 [0.16, 69.65]	1 [1]	8/234	8/347	1.48 [0.48, 4.6]	1 [0.45]
Joint pain	3/215	4/332	1.16 [0.17, 6.92]	1 [1]	3/130	10/717	1.65 [0.29, 6.54]	1 [0.436]	11/440	2/130	1.62 [0.35, 15.26]	1 [0.742]	24/219	50/306	0.67 [0.38, 1.15]	0.889 [0.132]
Wheezing	0/218	1/334	0 [0, 59.88]	1 [1]	1/130	1/729	5.59 [0.07, 439.12]	0.86 [0.281]	4/449	0/132	∞ [0.19, ∞]	1 [0.579]	3/239	5/350	0.88 [0.14, 4.57]	1 [1]
Cough	0/217	1/334	0 [0, 60.15]	1 [1]	1/131	0/723	∞ [0.14, ∞]	0.86 [0.154]	1/452	1/130	0.29 [0, 22.75]	1 [0.399]	8/235	13/342	0.9 [0.32, 2.37]	1 [1]

Fever	0/212	0/328	0<br>[0, ∞]	1<br>[1]	0/127	0/705	0<br>[0, ∞]	1<br>[1]	0/439	0/127	0<br>[0, ∞]	1<br>[1]	0/240	0/349	0<br>[0, ∞]	1<br>[1]
Headache	0/219	0/336	0<br>[0, ∞]	1<br>[1]	0/133	0/728	0<br>[0, ∞]	1<br>[1]	1/452	0/132	∞<br>[0.01, ∞]	1<br>[1]	8/237	12/344	0.97<br>[0.34, 2.62]	1<br>[1]
Chest pain	0/218	0/335	0<br>[0, ∞]	1<br>[1]	0/131	0/730	0<br>[0, ∞]	1<br>[1]	0/453	1/131	0<br>[0, 11.36]	1<br>[0.226]	1/241	4/351	0.36<br>[0.01, 3.71]	1<br>[0.653]
Ear pain	0/218	0/335	0<br>[0, ∞]	1<br>[1]	0/133	0/732	0<br>[0, ∞]	1<br>[1]	0/454	0/131	0<br>[0, ∞]	1<br>[1]	1/243	1/354	1.46<br>[0.02,<br>114.57]	1<br>[1]
Lymph nodes inflammation	0/218	0/335	0<br>[0, ∞]	1<br>[1]	0/133	1/731	0<br>[0, 213.93]	1<br>[1]	0/454	0/131	0<br>[0, ∞]	1<br>[1]	0/244	2/353	0<br>[0, 7.75]	1<br>[0.516]
Epilepsy	0/219	0/336	0<br>[0, ∞]	1<br>[1]	0/133	0/728	0<br>[0, ∞]	1<br>[1]	0/453	0/132	0<br>[0, ∞]	1<br>[1]	0/245	0/356	0<br>[0, ∞]	1<br>[1]
Sore throat	0/218	0/335	0<br>[0, ∞]	1<br>[1]	0/133	0/732	0<br>[0, ∞]	1<br>[1]	0/454	0/131	0<br>[0, ∞]	1<br>[1]	2/242	5/350	0.58<br>[0.06, 3.57]	1<br>[0.706]

Supplementary table 7. Odds, odds ratios with confidence intervals, adjusted and unadjusted Fisher's test p-values for individual symptom comparisons between SARS-CoV-2 males and females in infected and in non-infected groups at CON-VINCE visit 8.

Abbreviations: S+ = with persistent newly developed symptoms; S- = without persistent symptoms; M = males; F = females. OR = odds ratio; 95% CI = confidence interval 95%. * Indicates significant p-values at 5% confidence level before Benjamini-Hochberg multiple testing adjustment.

	**SARS-CoV-2 infected**				**SARS-CoV-2 non-infected**			
**Symptom**	M S+ / M S-	F S+ / F S-	OR [95% CI]	Adjusted p-value [p-value]	M S+ / M S-	F S+ / F S-	OR [95% CI]	Adjusted p-value [p-value]
**Depression**	10/99	2/98	4.95 [1.01, 47.3]	**0.677 [0.035*]**	7/151	10/167	0.77 [0.24, 2.32]	1 [0.804]
Loss of taste / smell	0/113	4/99	0 [0, 1.36]	0.677 [0.050]	0/158	0/175	0 [0, ∞]	1 [1]
Memory impairment	9/105	14/90	0.55 [0.20, 1.45]	1 [0.194]	11/148	13/165	0.94 [0.37, 2.36]	1 [1]
**Sleep disorders**	1/113	4/100	0.22 [0, 2.30]	1 [0.195]	1/157	10/165	0.1 [0, 0.76]	**0.311 [0.012*]**
Hair loss	0/113	2/101	0 [0, 4.84]	1 [0.226]	0/158	0/177	0 [0, ∞]	1 [1]
Shortness of breath	0/112	2/102	0 [0, 4.93]	1 [0.231]	0/157	3/173	0 [0, 2.7]	1 [0.250]
Fatigue	2/111	5/98	0.35 [0.03, 2.23]	1 [0.262]	2/157	4/171	0.54 [0.05, 3.87]	1 [0.687]
Muscle pain	2/111	4/99	0.45 [0.04, 3.20]	1 [0.428]	1/158	3/172	0.36 [0.01, 4.58]	1 [0.624]
Nausea/ vomiting / vertigo	0/114	1/103	0 [0, 35.58]	1 [0.477]	1/158	0/177	∞ [0.03, ∞]	1 [0.473]
Loss of appetite	0/113	1/102	0 [0, 35.55]	1 [0.477]	0/158	0/175	0 [0, ∞]	1 [1]
Mental confusion	0/113	1/103	0 [0, 35.89]	1 [0.479]	0/159	0/175	0 [0, ∞]	1 [1]
Anxiety	4/105	6/94	0.6 [0.12, 2.61]	1 [0.525]	9/149	11/166	0.91 [0.32, 2.50]	1 [1]
Arrhythmia / palpitations	1/112	1/103	0.92 [0.01, 72.84]	1 [1]	1/157	1/175	1.11 [0.01, 87.93]	1 [1]
Skin rash	1/113	1/102	0.9 [0.01, 71.50]	1 [1]	0/158	2/175	0 [0, 5.96]	1 [0.500]
Runny nose	1/111	1/103	0.93 [0.01, 73.50]	1 [1]	0/158	0/175	0 [0, ∞]	1 [1]

Joint pain	2/111	1/102	1.84 [0.09, 109.40]	1 [1]	1/158	3/172	0.36 [0.01, 4.58]	1 [0.624]
Diarrhoea	0/114	0/104	0 [0, ∞]	1 [1]	1/158	1/176	1.11 [0.01, 87.87]	1 [1]
Cough	0/113	0/102	0 [0, ∞]	1 [1]	1/157	0/175	∞ [0.03, ∞]	1 [0.474]
Abdominal pain	0/114	0/104	0 [0, ∞]	1 [1]	0/159	2/175	0 [0, 5.92]	1 [0.500]
Wheezing	0/112	0/104	0 [0, ∞]	1 [1]	0/157	1/175	0 [0, 43.69]	1 [1]
Fever	0/109	0/101	0 [0, ∞]	1 [1]	0/152	0/174	0 [0, ∞]	1 [1]
Headache	0/113	0/104	0 [0, ∞]	1 [1]	0/159	0/175	0 [0, ∞]	1 [1]
Chest pain	0/112	0/104	0 [0, ∞]	1 [1]	0/157	0/176	0 [0, ∞]	1 [1]
Ear pain	0/112	0/104	0 [0, ∞]	1 [1]	0/158	0/175	0 [0, ∞]	1 [1]
Lymph nodes inflammation	0/112	0/104	0 [0, ∞]	1 [1]	0/158	0/175	0 [0, ∞]	1 [1]
Epilepsy	0/113	0/104	0 [0, ∞]	1 [1]	0/159	0/175	0 [0, ∞]	1 [1]
Sore throat	0/112	0/104	0 [0, ∞]	1 [1]	0/158	0/175	0 [0, ∞]	1 [1]

Supplementary table 8. Odds, odds ratios with confidence intervals, adjusted and unadjusted Fisher's test p-values for individual comorbidities comparisons between individuals with and without persistent symptoms at the different study visits.

Abbreviations: S+ = with persistent newly developed symptoms; S- = without persistent symptoms; C+ = presence of the comorbidity at baseline. C- = absence of the comorbidity at baseline. OR = odds ratio; 95% CI = confidence interval 95%; * Indicates significant p-values at 5% confidence level before Benjamini-Hochberg multiple testing adjustment; *** significant after Benjamini-Hochberg multiplicity adjustment.

	**CON-VINCE V8 [MAIN ANALYSIS]**				CON-VINCE V7				CON-VINCE V9				CON-VINCE V8 UNADJUSTED FOR SYMPTOMS AT BASELINE			
**Comorbidity**	S+C+ / S-C+	S+C- / S-C-	OR [95% CI]	Adj. p-value [p-value]	S+C+ / S-C+	S+C- / S-C-	OR [95% CI]	Adj. p-value [p-value]	S+C+ / S-C+	S+C- / S-C-	OR [95% CI]	Adj. p-value [p-value]	S+C+ / S-C+	S+C- / S-C-	OR [95% CI]	Adj. p-value [p-value]
Other chronic diseases	34/74	82/369	2.06 [1.25, 3.38]	**0.052 [0.003*]**	29/138	114/585	1.08 [0.66, 1.71]	1 [0.729]	40/69	124/358	1.67 [1.05, 2.65]	**0.187 [0.024*]**	65/44	238/257	1.59 [1.03, 2.49]	**0.157 [0.034*]**
Chronic neurological disorder	9/9	105/430	4.08 [1.4, 11.92]	**0.052 [0.005*]**	6/21	135/694	1.47 [0.48, 3.85]	1 [0.427]	11/8	151/418	3.8 [1.36, 11.09]	**0.15 [0.007*]**	15/4	283/296	3.91 [1.23, 16.39]	**0.064 [0.01*]**
Rheumatological disorder	26/65	82/357	1.74 [1, 2.98]	**0.34 [0.044*]**	24/105	106/586	1.26 [0.74, 2.09]	1 [0.358]	30/58	118/359	1.57 [0.93, 2.62]	0.492 [0.086]	60/36	218/259	1.98 [1.23, 3.2]	**0.04*** [0.003*]**
Autoimmune disease	14/32	100/400	1.75 [0.83, 3.52]	0.73 [0.127]	14/60	127/646	1.19 [0.59, 2.23]	1 [0.624]	19/34	140/388	1.55 [0.81, 2.9]	0.684 [0.149]	33/16	261/281	2.22 [1.15, 4.42]	**0.064 [0.011*]**
Diabetes	3/29	107/407	0.39 [0.08, 1.31]	0.783 [0.17]	2/35	137/673	0.28 [0.03, 1.12]	0.534 [0.07]	5/17	152/408	0.79 [0.22, 2.28]	1 [0.808]	18/16	276/281	1.15 [0.54, 2.45]	1 [0.727]
Asthma	11/26	104/417	1.69 [0.73, 3.69]	0.783 [0.204]	10/31	133/688	1.67 [0.71, 3.59]	1 [0.194]	9/26	152/400	0.91 [0.37, 2.06]	1 [1]	21/17	280/284	1.25 [0.61, 2.59]	1 [0.616]
Mild liver disease	1/14	112/422	0.27 [0.01, 1.81]	0.845 [0.327]	0/23	138/686	0 [0, 0.88]	**0.258 [0.022*]**	4/12	156/408	0.87 [0.2, 2.93]	1 [1]	8/7	286/292	1.17 [0.36, 3.83]	1 [0.8]

Non-chronic cardiovascular disease	8/21	107/418	1.49 [0.55, 3.61]	0.845 [0.35]	8/32	133/685	1.29 [0.5, 2.93]	1 [0.514]	12/26	150/397	1.22 [0.55, 2.58]	1 [0.577]	17/15	280/286	1.16 [0.53, 2.54]	1 [0.72]
Chronic haematological disease	0/7	116/434	0 [0, 2.64]	0.845 [0.354]	1/12	142/708	0.42 [0.01, 2.85]	1 [0.706]	2/5	161/421	1.05 [0.1, 6.47]	1 [1]	3/4	297/297	0.75 [0.11, 4.48]	1 [1]
Chronic kidney disease	1/1	113/442	3.9 [0.05, 306.93]	0.845 [0.368]	1/5	141/712	1.01 [0.02, 9.12]	1 [1]	2/4	158/422	1.33 [0.12, 9.42]	1 [0.667]	2/1	299/300	2 [0.1, 118.7]	1 [1]
Psychiatric disease	6/16	108/422	1.46 [0.46, 4.06]	0.845 [0.424]	13/26	129/686	2.65 [1.22, 5.52]	**0.174 [0.008*]**	9/13	154/409	1.84 [0.68, 4.75]	0.753 [0.223]	19/4	277/296	5.06 [1.65, 20.71]	**0.027*** [0.001*]**
Immuno-suppression	3/7	113/435	1.65 [0.27, 7.36]	0.845 [0.441]	4/13	139/709	1.57 [0.37, 5.18]	1 [0.504]	6/8	158/418	1.98 [0.56, 6.63]	0.753 [0.229]	6/5	297/295	1.19 [0.3, 4.99]	1 [1]
Severe liver disease	1/2	114/436	1.91 [0.03, 37]	0.891 [0.504]	0/3	142/706	0 [0, 12.12]	1 [1]	1/2	159/420	1.32 [0.02, 25.52]	1 [1]	1/2	297/297	0.5 [0.01, 9.66]	1 [1]
Hypertension	26/90	87/350	1.16 [0.68, 1.95]	0.993 [0.604]	22/132	119/584	0.82 [0.48, 1.36]	1 [0.473]	32/77	132/348	1.1 [0.67, 1.77]	1 [0.723]	63/58	233/243	1.13 [0.74, 1.72]	1 [0.543]
Stroke	2/6	112/436	1.3 [0.13, 7.38]	1 [0.67]	2/9	140/708	1.12 [0.12, 5.51]	1 [0.701]	1/6	163/419	0.43 [0.01, 3.58]	1 [0.68]	4/4	296/296	1 [0.18, 5.42]	1 [1]
Chronic pulmonary disease	4/13	112/427	1.17 [0.27, 3.89]	1 [0.764]	4/16	138/702	1.27 [0.3, 4.02]	1 [0.759]	6/11	157/413	1.43 [0.43, 4.32]	1 [0.582]	8/9	293/291	0.88 [0.29, 2.62]	1 [0.812]
Chronic cardiac disease	4/18	109/414	0.84 [0.2, 2.64]	1 [1]	5/26	131/680	1 [0.29, 2.71]	1 [1]	6/14	155/402	1.11 [0.34, 3.15]	1 [0.803]	13/10	280/283	1.31 [0.52, 3.41]	1 [0.671]
Cancer	9/34	107/407	1.01 [0.41, 2.23]	1 [1]	10/49	133/667	1.02 [0.45, 2.11]	1 [1]	19/25	144/399	2.1 [1.06, 4.11]	**0.187 [0.023*]**	26/19	275/282	1.4 [0.73, 2.75]	1 [0.353]
HIV	1/3	114/438	1.28 [0.02, 16.11]	1 [1]	0/2	143/715	0 [0, 26.76]	1 [1]	0/1	162/424	0	1 [1]	3/1	298/299	3.01 [0.24, 158.41]	1 [0.624]

											[0, 102.15]					
Organ transplantation	0/1	116/442	0 [0, 148.6]	1 [1]	0/2	143/721	0 [0, 26.98]	1 [1]	0/4	164/423	0 [0, 3.95]	1 [0.58]	1/2	302/299	0.5 [0.01, 9.57]	1 [0.623]
Dementia	0/1	116/442	0 [0, 148.6]	1 [1]	0/0	143/720	0 [0, ∞]	1 [1]	0/0	164/427	0 [0, ∞]	1 [1]	1/0	302/301	∞ [0.03, ∞]	1 [1]
Malnutrition	0/0	114/441	0 [0, ∞]	1 [1]	0/1	142/717	0 [0, 196.59]	1 [1]	0/0	161/424	0 [0, ∞]	1 [1]	0/0	298/301	0 [0, ∞]	1 [1]
Neurodegenerative disease	0/2	115/441	0 [0, 20.57]	1 [1]	0/1	142/718	0 [0, 196.87]	1 [1]	1/0	160/427	∞ [0.07, ∞]	0.787 [0.274]	2/0	300/301	∞ [0.19, Inf]	1 [0.499]

Supplementary table 9. Odds, odds ratios with confidence intervals, adjusted and unadjusted Fisher's test p-values for individual comorbidities comparisons between SARS-CoV-2 infected and non-infected individuals at CON-VINCE visit 8.

Abbreviations: S+ = with persistent newly developed symptoms; S- = without persistent symptoms; C+ = presence of the comorbidity at baseline. C- = absence of the comorbidity at baseline. OR = odds ratio; 95% CI = confidence interval 95%. * significant before Benjamini-Hochberg multiplicity adjustment.

Comorbidity	Infected				Non-infected			
	S+C+ / S-C+	S+C- / S-C-	OR [95% CI]	Adjusted p-value [p-value]	S+C+ / S-C+	S+C- / S-C-	OR [95% CI]	Adjusted p-value [p-value]
Other chronic diseases	14/36	29/141	1.88 [0.83, 4.15]	0.871 [0.105]	20/46	45/228	2.20 [1.12, 4.22]	**0.111 [0.014*]**
Autoimmune disease	6/44	9/158	2.38 [0.66, 7.97]	0.871 [0.118]	8/56	23/242	1.50 [0.55, 3.71]	1 [0.345]
Asthma	5/45	8/162	2.24 [0.55, 8.22]	0.871 [0.177]	6/59	18/255	1.44 [0.45, 4]	1 [0.428]
Non-chronic cardiovascular disease	5/45	8/161	2.23 [0.55, 8.17]	0.871 [0.179]	3/62	13/257	0.96 [0.17, 3.63]	1 [1]
Diabetes	0/47	8/160	0 [0, 2.08]	0.871 [0.205]	3/60	21/247	0.59 [0.11, 2.07]	1 [0.589]
Chronic kidney disease	1/49	0/170	∞ [0.09, Inf]	0.871 [0.227]	0/64	1/272	0 [0, 165.93]	1 [1]
Cancer	4/46	7/162	2.01 [0.41, 8.30]	0.914 [0.278]	5/61	27/245	0.74 [0.21, 2.07]	1 [0.647]
Immunosuppression	1/49	1/168	3.4 [0.04, 270.09]	1 [0.405]	2/64	6/267	1.39 [0.13, 8.00]	1 [0.656]
Psychiatric disease	3/46	6/163	1.77 [0.28, 8.66]	1 [0.424]	3/62	10/259	1.25 [0.22, 5.06]	1 [0.723]
Mild liver disease	0/49	4/163	0 [0, 5.19]	1 [0.576]	1/63	10/259	0.41 [0.01, 2.99]	1 [0.698]
Chronic neurological disorder	2/48	4/166	1.72 [0.15, 12.45]	1 [0.621]	7/57	5/264	6.43 [1.69, 26.67]	**0.058 [0.003*]**
Hypertension	10/40	30/139	1.16 [0.46, 2.70]	1 [0.683]	16/47	60/211	1.2 [0.59, 2.33]	1 [0.617]
Rheumatological disorder	6/42	18/144	1.14 [0.35, 3.25]	1 [0.798]	20/40	47/213	2.26 [1.14, 4.38]	**0.111 [0.013*]**
Chronic pulmonary disease	1/49	5/164	0.67 [0.01, 6.20]	1 [1]	3/63	8/263	1.56 [0.26, 6.75]	1 [0.456]
Chronic cardiac disease	2/47	7/159	0.97 [0.09, 5.31]	1 [1]	2/62	11/255	0.75 [0.08, 3.56]	1 [1]
Chronic haematological disease	0/50	3/165	0 [0, 8.19]	1 [1]	0/66	4/269	0 [0, 6.3]	1 [1]
Stroke	0/50	2/168	0 [0, 18.20]	1 [1]	2/62	4/268	2.16 [0.19, 15.43]	1 [0.321]

Neurodegenerative disease	0/50	1/169	0 [0, 132.33]	1 [1]	0/65	1/272	0 [0, 163.38]	1 [1]
HIV	0/49	1/169	0 [0, 135.02]	1 [1]	1/65	2/269	2.06 [0.03, 40.21]	1 [0.481]
Severe liver disease	0/49	1/167	0 [0, 133.44]	1 [1]	1/65	1/269	4.11 [0.05, 325.14]	1 [0.355]
Dementia	0/50	0/170	0 [0, ∞]	1 [1]	0/66	1/272	0 [0, 160.92]	1 [1]
Malnutrition	0/49	0/170	0 [0, ∞]	1 [1]	0/65	0/271	0 [0, ∞]	1 [1]
Organ transplantation	0/50	0/170	0 [0, ∞]	1 [1]	0/66	1/272	0 [0, 160.92]	1 [1]

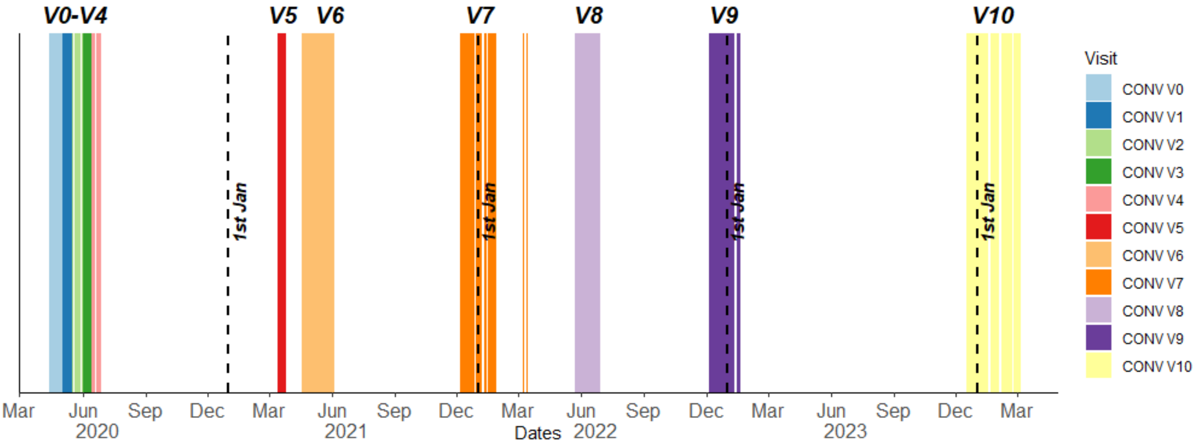

Supplementary figure 1. CON-VINCE / ORCHESTRA Europe study visits distribution of dates. V0-V6 visits were conducted within the CON-VINCE framework, while V7-V10 visits were conducted under ORCHESTRA Luxembourg.

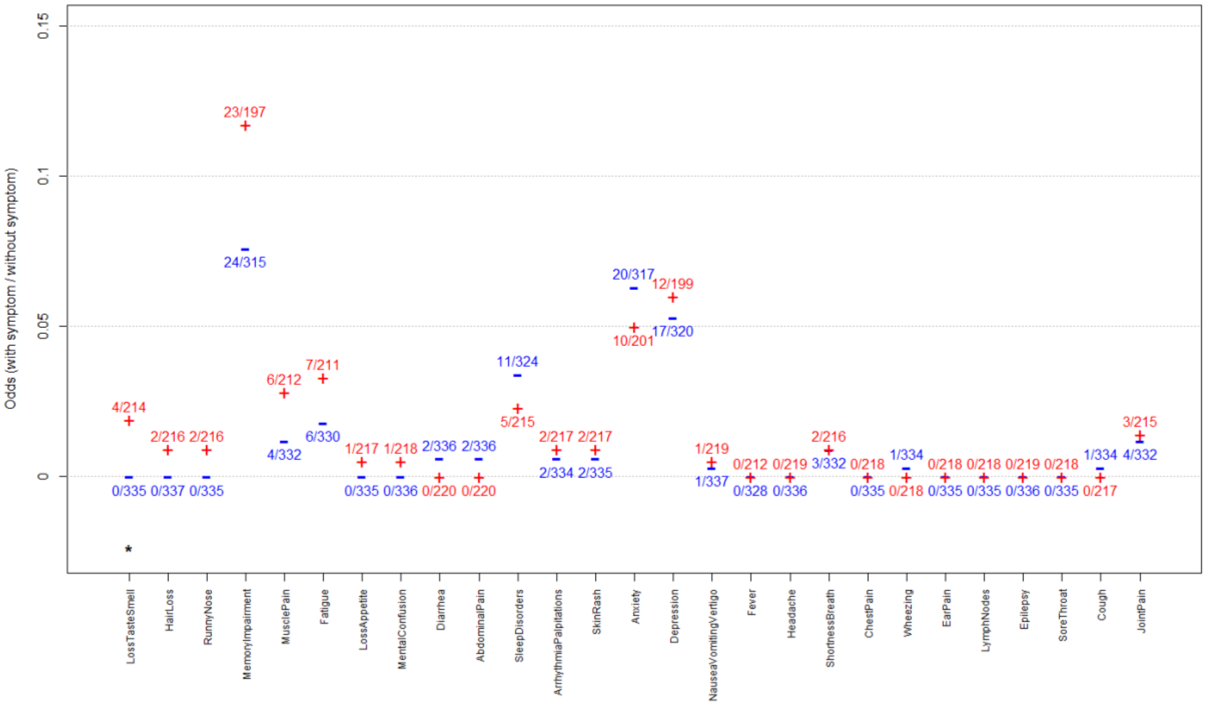

Supplementary figure 2. Odds of 27 symptoms in all SARS-CoV-2 infected and non-infected participants at CON-VINCE visit 8.

The values represent the number of individuals with the persistent symptom divided by the number of individuals without the persistent symptom in the subgroup. The infected subgroup is represented in red, and the non-infected one in blue. * Significantly different symptoms between the subgroups **before** adjustment for multiplicity at 5% confidence level.

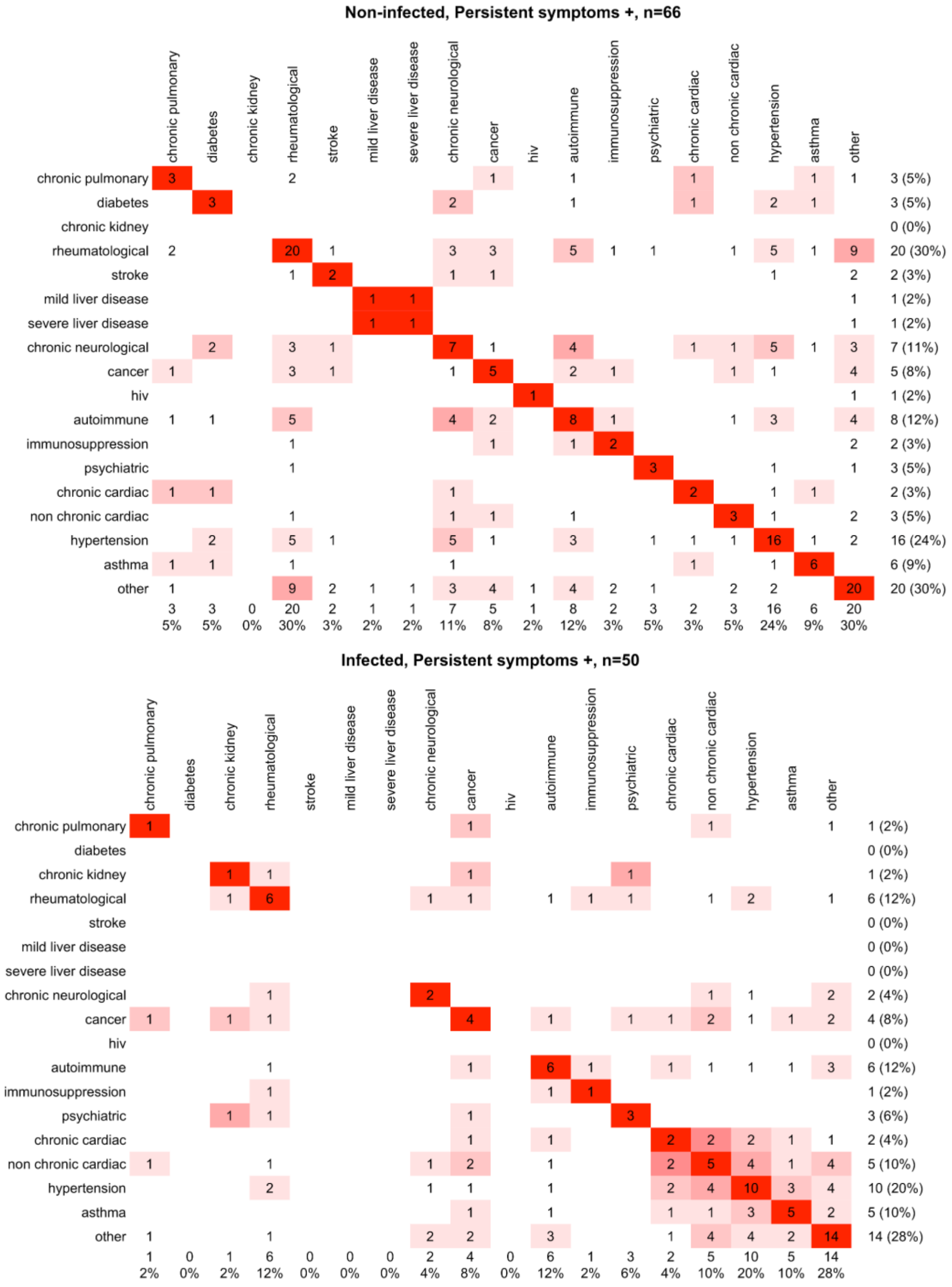

Supplementary figure 3. Pairwise combinations of comorbidities in SARS-CoV-2 infected individuals (top) and in non-infected individuals (bottom) reporting at least one persistent symptom at CON-VINCE visit 8.

Heat maps are based on the Jaccard similarity index between comorbidities. The Jaccard index ranges from zero for dissimilar (white) to one for similar (dark red), and it is not defined if both comorbidities never occur. The numbers on the right and on the bottom indicate the number of individuals with the given comorbidity, with the proportion of individuals with this comorbidity in the considered subgroup in brackets. Only comorbidities self-reported by at least one participant are displayed.

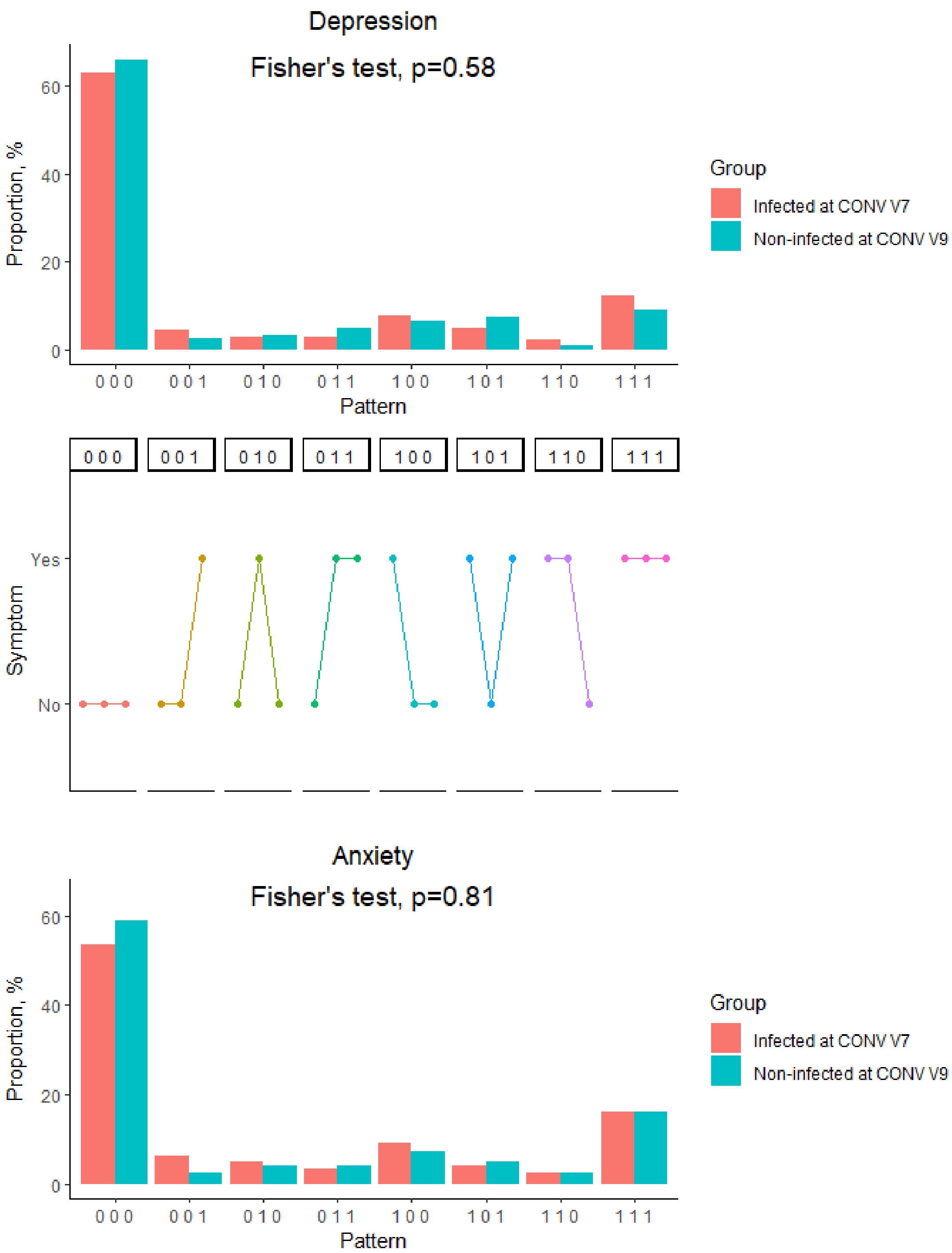

Supplementary figure 4. Longitudinal trajectories of depression and anxiety, stratified by SARS-CoV-2 infection status.

The patterns consist of 0s and 1s, where 0 indicates that an individual does not have Depression (top) / Anxiety (bottom) at a given visit, while 1 indicates that Depression (top) / Anxiety (bottom) was present at the visit. The infected group at CON-VINCE visit 7 was compared to the group that stayed non-infected until CON-VINCE visit 9. Only individuals with available data for all the three time points (visits 7, 8, 9 of CON-VINCE) were included in the analysis.
